# Induced Neural Progenitor Specification from Human Pluripotent Stem Cells by a Refined Synthetic Notch Platform

**DOI:** 10.1021/acssynbio.4c00742

**Published:** 2025-05-06

**Authors:** Catherine A. Hamann, Andrew Kjar, Hyosung Kim, Alan J. Simmons, Hannah J. Brien, Cheryl I. Quartey, Bonnie L. Walton, Ken S. Lau, Ethan S. Lippmann, Jonathan M. Brunger

**Affiliations:** † Department of Biomedical Engineering, Vanderbilt University, Nashville, Tennessee 37235, United States; ‡ Department of Chemical and Biomolecular Engineering, Vanderbilt University, Nashville, Tennessee 37235, United States; § Department of Cell and Developmental Biology, Vanderbilt University, Nashville, Tennessee 37235, United States; ∥ Department of Biological Sciences, Vanderbilt University, Nashville, Tennessee 37235, United States; ⊥ Center for Stem Cell Biology, Vanderbilt University, Nashville, Tennessee 37235, United States; # Center for Computational Systems Biology, Vanderbilt University, Nashville, Tennessee 37235, United States

**Keywords:** synthetic biology, synthetic morphogenesis, central nervous system, Sonic Hedgehog, floor plate, synNotch

## Abstract

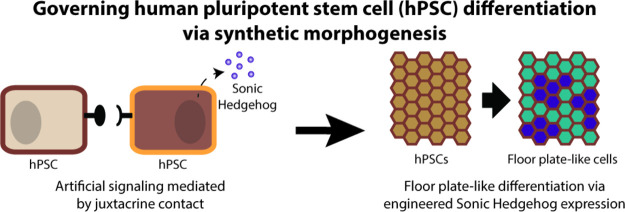

Historically, studying the development of brain and central nervous system (CNS) tissues has been challenging. Human pluripotent stem cell (hPSC) technology has allowed for the in vitro reconstitution of relevant, early cell trajectories by using small molecules and recombinant proteins to guide differentiation of cells toward relevant brain and CNS phenotypes. However, many of these protocols fail to recapitulate the cell-guided differentiation programs intrinsic to embryonic development, particularly the signaling centers that emerge within the neural tube during brain formation. Located on the ventral end of the neural tube, the floor plate acts as one such signaling center to pattern the dorsal/ventral axis by secreting the morphogen Sonic Hedgehog (SHH). Here, we present a method for cell-guided differentiation using the synthetic Notch (synNotch) receptor platform to regulate SHH production and subsequent cell fate specification. We show that the widely used configuration of the orthogonal synNotch ligand green fluorescent protein (GFP) mounted on a platelet-derived growth factor receptor-β transmembrane chassis does not allow for robust artificial signaling in synNotch-hPSCs (“receivers”) cocultured with ligand-presenting hPSCs (“senders”). We discovered that refined designs of membrane-bound GFP-ligand allow for efficient receptor activation in hPSC receivers. A variant of this enhanced synNotch system drives the production of SHH in hPSC sender:hPSC receiver cocultures and gives rise to floor plate-like cell types seen during neural tube development. This revised synNotch platform has the potential to pattern hPSC differentiation programs in synthetic morphogenesis studies designed to uncover key paradigms of human CNS development.

## Introduction

Understanding early embryogenesis, particularly that of the central nervous system (CNS), proves to be difficult, in part due to the brain and CNS taking shape during incredibly nascent stages of fetal growth around the third and fourth week of gestation.^1^ Nonetheless, discerning these complex processes can lend insight into the structural organization of tissues, the subtle perturbation of which often gives rise to diseases and disorders, including spina bifida^2−4^ and anencephaly.^2,5^ Thus, there is a tremendous need to learn how to establish cell fates of the CNS, both to determine developmental paradigms of the human CNS and to establish cell sources for prospective cell-based therapies and tissue engineering strategies.

Despite major challenges in understanding early brain organogenesis, there is a growing appreciation that coregulated expression of morphogens and their antagonists from within specific and transient tissues governs the emergence of brain domains from the neural tube, the precursor of the CNS. For example, approximately 20–30 days following fertilization, polarized regions known as cell signaling centers secrete morphogens that give rise to distinct cell types that later form the hindbrain, midbrain, and forebrain.^6^ One such center, the floor plate, is located on the ventral end of the neural tube and supplies the morphogen Sonic Hedgehog (SHH).^7−9^ SHH patterns ventral neural progenitors that go on to form more mature cell types, including midbrain dopaminergic neurons^10^ and oligodendrocyte progenitor cells.^11^ While tremendous effort has focused on deriving highly specialized CNS cells from human pluripotent stem cells (hPSCs), only few studies have aimed to establish methods that recapitulate the cell:cell signaling and morphogen secretion that orchestrate production of signaling centers such as the floor plate.^12,13^ Achieving this not only requires protocols for directing hPSCs to target fates, but also necessitates the design of artificial signaling modalities that mimic native cell:cell interactions to instruct the formation of such signaling centers.

**Figure 1:**
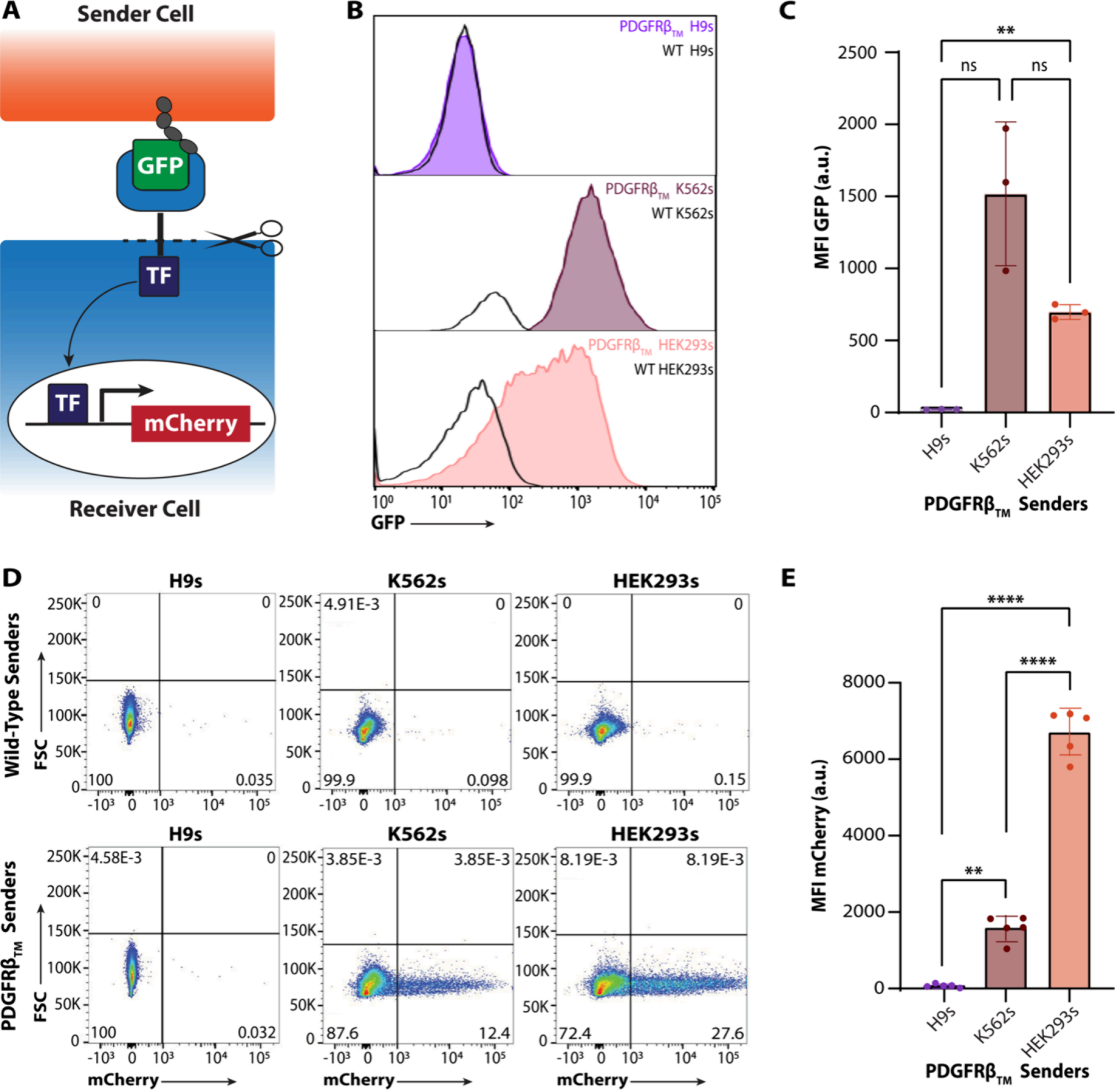
Canonical synNotch ligand mounted on a PDGFRβ_TM_ motif is not effective in H9 senders but is competent to activate H9 receivers when presented by non-PSC sender cells. (A) A juxtacrine platform, synNotch consists of a sender cell presenting a ligand, such as GFP, using a truncated PDGFRβ_TM_ protein. Neighboring receiver cells express a cognate receptor that incorporates a GFP-sensitive nanobody, which can bind to the ligand on sender cells, resulting in a conformation change that causes cleavage at the transmembrane core and translocation of a transcription factor (TF) to the nucleus. The TF then activates user-defined transgene expression, such as expression of the fluorescent reporter mCherry. (B,C) Flow cytometry results of overall expression of PDGFRβ_TM_ H9s, K562s, and HEK293s. Welch’s ANOVA with Tukey’s multiple comparisons post hoc: ns: not statistically significant, **p < 0.01. (D,E) Flow cytometry results after 72-h cocultures of 1:1 ratio of mCherry H9 receivers and senders from each WT or PDGFRβ_TM_ cell type. Welch’s ANOVA with Tukey’s multiple comparisons post hoc: **p < 0.01, ****p < 0.0001.

The field of synthetic morphogenesis seeks to develop a rules-based means whereby desired biological architectures can be assigned through engineering principles. A targeted objective of this field is to harness the ability to coordinate tissue formation, regeneration, and repair. Toward these goals, elegant synthetic morphogenesis efforts have been made to mimic neurodevelopment, including those that employ drug-inducible systems,^12,14,15^ optogenetic platforms,^13,14,16,17^ biomaterial-mediated signaling,^18−20^ and artificial receptor modules.^20^ One platform that has received wide adoption is the synthetic Notch^21^ (synNotch) signaling channel. Modeled after native Notch, synNotch receptors respond to defined and immobilized juxtacrine cues, such as transmembrane ligands on neighboring cells, to activate expression of transgenes downstream of an artificial promoter regulated by synNotch activity (Figure 1A). The requirement for ligand immobilization leads to highly localized expression of customized transgenes. SynNotch has been used with a number of different cell types in the area of synthetic morphogenesis, including L929 mouse fibroblasts,^21−25^ human epithelial HEK293 cells,^21,26,27^ and mouse ESCs^28^ to regulate complex multicellular activities, such as self-organization of varied cell types in spheroids,^23^ emergent stripe patterning in response to captured soluble ligands,^24^ and stem cell differentiation toward neuronal lineages.^28^ The highly modular nature of synNotch is especially attractive, because it is orthogonal to native signaling pathways commonly active in development and enables selected input-to-output pairing, all while functioning in a biomimetic manner by relying on cell:cell juxtacrine interactions. However, to date there has been no use of synNotch with hPSCs in a juxtacrine manner, only the use of synNotch-engineered hPSCs with a ligand-presenting biomaterial surface.^20,22,25^ An operational synNotch toolkit that can be used to engineer hPSCs and function in a juxtacrine context would prove incredibly useful, allowing for user-specified transgene expression through ligand:receptor interactions that may enable bottom-up mimicry of developmental events of interest in the domain of synthetic morphogenesis.^29^

Here, we deploy synNotch in synthetic morphogenesis studies to orchestrate the emergence of human floor plate progenitors from hPSCs. We found that the prototypical orthogonal synNotch ligand, green fluorescent protein (GFP) mounted on a platelet-derived growth factor receptor-β transmembrane (PDGFRβ_TM_) chassis, is ineffective in hPSCs. We then identified multiple alternative functional ligand chassis and moved forward with one of these improved ligand variants in juxtacrine hESC sender:receiver experiments. These studies interrogated the ability of synNotch to guide specification of floor plate progenitors in synNotch-hESC sender:receiver cocultures. Our molecular analyses reveal that while synNotch cocultures exhibit greater heterogeneity than cells given recombinant SHH, they still generate floor plate-like cells with similar gene expression profiles, underscoring the ability of synNotch-mediated SHH induction to specify floor plate identity. Our studies provide critical guidance on how to apply the synNotch platform in hPSC synthetic morphogenesis studies and indicate the potential for using the synNotch artificial signaling channel to derive floor plate progenitors from hPSCs.

## Materials and Methods

### SynNotch Constructs

Sleeping Beauty (SB) transposon plasmids (based on Addgene 60495, a kind gift from Eric Kowarz)^30^ for both sender and receiver constructs were designed in Snapgene and cloned using NEBuilderⓇ HiFi DNA Assembly. Sender constructs containing Epithelial Cadherin (E-Cadherin) and GFP were based on Addgene 205188, a kind gift from Wendell Lim.^31^ Those with Duffy Antigen for Chemokines (DARC) and GFP were based on Addgene 105206, a kind gift from Timothy Lu.^32^ Following assembly, plasmids were transformed into NEB5α *E. coli* competent cells and plated on LB agar plates supplemented with ampicillin for overnight incubation at 37 °C. Colonies were picked and cultured in LB broth supplemented with ampicillin and placed at 37 °C shaking at 250 rpm overnight before performing miniprep purification (Qiagen 27104). All plasmids were verified via Sanger sequencing.

### HEK293 Cells and Culture Conditions

HEK293s were cultured in DMEM with 10% fetal bovine serum (FBS). Once cells reached 70% confluency, a reverse transfection was performed at a 1:1 ratio with the SB 100× transposase (Addgene 34879, a kind gift from Zsuzsanna Izsvak)^33^ and the SB transposon encoding the ligand-presenting sender cell synNotch constructs using the Mirus *Trans*ITⓇ-LT1 Transfection Reagent (MIR 2304). Cells were selected in 4 μg/mL puromycin for roughly 1 week to enrich transfected cells before being sorted for positive GFP expression with a 4-laser FACSAria III and used in subsequent experiments.

### K562 Cells and Culture Conditions

K562s were cultured in IMDM with 10% FBS. Once cells reached approximately 2 million cells/mL, a standard transfection was performed at a 1:1 ratio with the SB transposase vector (Addgene 34879, a kind gift from Zsuzsanna Izsvak)^33^ and the SB transposon encoding the ligand-presenting sender cell synNotch constructs using Lipofectamine 3000 (Invitrogen L3000001). Cells were selected in 2.5 μg/mL puromycin for roughly 1 week to enrich transfected cells before being sorted for positive GFP expression as previously described and used in subsequent experiments.

### H9 hESC Culture Conditions

H9 cells used in these studies were validated via short tandem repeat (STR) fingerprinting (ATCC) and tested negative for mycoplasma (Bulldog Bio 2523148). They were cultured in mTeSR Plus (STEMCELL TECHNOLOGIES 100-1130) on Geltrex (Gibco A1413201)-coated tissue culture plates. Once 70% confluent, cells were passaged using Accutase along with mTeSR Plus supplemented with 10 μM Y-27632 (Tocris 1254). A reverse transfection was performed with 1.2E6 cells/mL at a 1:1 ratio with the SB transposase (Addgene 34879, a kind gift from Zsuzsanna Izsvak)^33^ and the SB transposon encoding either the GFP-responsive LaG16-synNotch receptor or a variant of the GFP synNotch ligand using the Mirus *Trans*ITⓇ-LT1 Transfection Reagent (MIR 2304). Cells were selected in 1.2 μg/mL puromycin for approximately 1 week to enrich transfected cells before being sorted for either synNotch receptor expression or GFP expression. Sender cells expressing a GFP-ligand either on the extracellular surface of E-Cadherin (Ecad H9s) or on the DARC domain (DARC H9s) were sorted such that they expressed the same level of GFP. Sender cells expressing a GFP-ligand on the PDGFRβ_TM_ domain (PDGFRβ_TM_ H9s) had GFP expression levels similar to wild-type (WT) hESCs and were therefore not sorted. To sort synNotch receiver cells expressing either mCherry (mCherry H9s) or SHH (SHH H9s), receiver hESCs were stained using a fluorescently labeled Myc-Tag antibody (Cell Signaling 2233S) to identify myc-tagged synNotch expression. Myc+ receiver hESCs were used for subsequent experiments.

### GFP Flow Cytometry

HEK293s and H9 hESCs were dissociated using Accutase and K562s were collected in suspension before being centrifuged at 300×g for 5 min and resuspended in PBS supplemented with 5% FBS. Total GFP expression was examined using a Cytek Guava easyCyte Flow Cytometer and analyzed using FlowJo. All flow cytometry measures of GFP fluorescence intensity capture both intracellular and membrane-bound, extracellular GFP levels.

### Flow Cytometry for H9 mCherry Readout of synNotch Activation

At the second passage from thaw, HEK293:H9 hESC, K562:H9 hESC, and H9 hESC:H9 hESC cocultures were plated at a density of 400,000 cells/cm^2^ in 96-well plates coated with Geltrex. Cocultures were seeded at a 1:1 ratio of senders:receivers in mTeSR Plus supplemented with 10 μM Y-27632. Twenty-four h after plating, medium was changed and replaced with mTeSR Plus excluding Y-27632. Seventy-two hours after plating, cocultures were dissociated using Accutase before being fixed with 4% paraformaldehyde for 10 min, washed with PBS, and permeabilized with permeabilization buffer (PBS containing 0.5% Triton-X100) for 10 min. After permeabilization, cells were blocked with blocking buffer (PBS containing 0.1% Triton-X100 and 5% FBS). The mCherry primary antibody (rabbit anti-mCherry [1:200; Cell Signaling 43590]) was applied for 1 h at room temperature before washing with PBS. The secondary antibody (AlexaFluor 647 donkey antirabbit [1:400; Invitrogen A31573]) was also applied for 1 h at room temperature protected from light before washing with PBS and placing cells in blocking buffer. Inducible mCherry expression was examined using a BD LSRFortessa Cell Analyzer and analyzed using FlowJo.

### Neural Induction and Floor Plate Specification

At the second passage from thaw, H9 hESCs were plated at a density of 400,000 cells/cm^2^ in 96-well plates coated with Geltrex. SynNotch conditions were seeded at a 1:1 ratio of senders:receivers. Neural induction medium was prepared on day 0 and contained neurobasal medium (Gibco, 21103049) supplemented with 1% N2 supplement (Gibco, 17502048), 2% B-27 supplement without vitamin A (Gibco, 12587010), 1% GlutaMAX (Gibco, 35050061), 10 μM SB431542 (STEMCELL TECH, 72234), 100 nM LDN193189 (STEMCELL TECH, 72149), and 10 μM Y-27632. WT H9 hESCs were also plated with 200 ng/mL SHH protein^34−36^ (Sonic C25II, R&D Systems, 464-SH). Medium was prepared (excluding Y-27632) and changed daily, and cells were taken out to 11 days in culture.

### Immunofluorescence and Imaging of Differentiated Cells

Differentiated H9 hESCs were fixed with 4% paraformaldehyde for 10 min, washed with PBS, and permeabilized with permeabilization buffer for 10 min. After permeabilization, cells were blocked with blocking buffer. Primary antibodies (goat anti-FOXA2 [1:200; R&D Systems AF2400] and rabbit anti-PAX6 [1:100; Biolegend 901301]) were applied for 1 h at room temperature before washing with PBS. Secondary antibodies (AlexaFluor 647 donkey antigoat [1:400; Invitrogen A-21447] and 488 goat antirabbit [1:400; Invitrogen A-32731]) were also applied for 1 h at room temperature protected from light before washing with PBS. Cells were also counterstained with DAPI (1:1000; Thermo Scientific 62247) before being washed and placed in blocking buffer. Cells were imaged using a Nikon Yokogawa CSU-XI spinning disk confocal microscope. Images were analyzed using Fiji ImageJ and converted to 8-bit. To determine the percentage FOXA2+ area along with the maximum fluorescence intensity values, background subtraction was performed for all images (300 rolling ball radius) before thresholding was performed for each channel. Area fraction and maximum gray values were determined for each image. For percentage FOXA2+ area, FOXA2 signal was normalized to corresponding DAPI images, where DAPI+ cells represented 100% area for each individual image.

### Bulk RNA Sequencing

Neural induction and floor plate specification was performed as described above and cells were taken out to 11 days in culture. mRNA was prepared using the PureLink RNA Mini Kit (ThermoFisher Scientific 12183018A) before handing off samples to Vanderbilt University Medical Center (VUMC) Vanderbilt Technologies for Advanced Genomics (VANTAGE). VANTAGE performed RNA quality control (QC) (with all samples achieving a QC assessment of 98% or higher) before moving toward stranded mRNA (NEB) library preparation and Novaseq X series PE150 sequencing. Raw sequencing reads were demultiplexed and converted to FASTQ format. High-quality reads were aligned to the human reference genome (Feb. 2022 GRCh38.p14) using HISAT2 (v2.2.1) with default parameters. Gene expression levels were quantified using featureCounts (v2.0.1) within the Subread package. Differential expression analysis was performed using EdgeR (v3.34.0) and limma (v3.46.0)^37^ R packages. Normalization of gene expression data was performed using the trimmed mean of M-values method implemented in EdgeR, followed by log-transformation for downstream analysis. Genes with a count per million >4 in at least one sample were retained for analysis, and differential expression was identified using a false discovery rate threshold of <0.05. Functional enrichment analysis was conducted using the WEB-based Gene Set Analysis Toolkit (WebGestalt).^38^ Transcript per million (TPM) values were calculated from raw counts by normalizing counts by gene length (in kilobases) and scaling by the sum of length-normalized counts per sample. The bulk RNA sequencing data generated in this study have been deposited in the NCBI Gene Expression Omnibus (GEO) under accession number GSE294935.

### Single-Cell RNA Sequencing

Neural induction and floor plate specification were performed as described above and cells were taken out to 11 days in culture. Cells were dissociated using Accutase before running particle-templated instant partition sequencing^39^ (Fluent Biosciences, PIPseq V4 T2 3′ Single Cell RNA Kit). Briefly, cells were coencapsulated with capture beads through vortexing. The resulting emulsion was then incubated to allow for lysis of cells and hybridization of mRNA to barcoded oligos on the beads. Beads were released from droplets and washed before undergoing reverse transcription and PCR reactions, resulting in amplified barcoded transcript libraries. Libraries were then fragmented, a-tailed and indexed for sequencing. Sequencing of the libraries was performed on NovaSeq6000. Reads were aligned using the pipseeker^39^ algorithm at a resolution of 5 and then read into Seurat (v5).^40^ Cells containing between 1600 and 7500 were retained. All data were merged, normalized, scaled based on the top 2000 variable features, and then scored for cell cycle using the default implementation in Seurat. Data were visualized using principal component analysis (PCA) and uniform manifold approximation and projection (UMAP). Clusters were assigned using the Leiden algorithm at a resolution of 1.5, and clusters of low-quality (low transcript) cells were detected and filtered out. Cleaned data were then reprocessed via PCA, UMAP, and Leiden clustering at a resolution of 1.5. The number of cells passing quality control (QC) were as follows: 5168 (WT H9s with recombinant SHH [WT (+)SHH]), 6542 (SHH H9s in coculture with WT H9s [SynNotch (−)GFP]), and 7222 (SHH H9s in coculture with Ecad H9s [SynNotch (+)GFP]), with *n* = 3 biological samples per culture condition. A floor plate module score was computed using the AddModuleScore function, based on the following four marker genes: *FOXA2*, *FOXA1*, *ARX*, and *TFF3*. Cells with a score greater than 0.25 were assigned as floor plate cells. All remaining cells were annotated per cluster based on gene expression as follows: *PAX6, OTX2, EMX2—*dorsal forebrain progenitors; *NKX2-1, RAX, SIX6—*ventral tuberal hypothalamic progenitors.^13^ To annotate cells based on single gene expression (*FOXA2*, *PAX6*, *NES*, *SOX2*), cells with an expression value greater than 0.25 were considered positive. To compare floor plate cells between samples, cells were aggregated by cell type and biological sample using the AggregateExpression function in Seurat. Then, differential genes were computed using the FindMarkers function and DESeq2 algorithm. The VoxHunt package was used to compare cell signatures to the BrainSpan atlas.^41^ Floor plate clusters were also extracted from previously published data^42^ and pseudobulked using the AggregateExpression function in Seurat. Then, pseudobulked cells from the previously published data and experimental conditions were compared to Pearson correlation coefficient or visualized using PCA. Code to reproduce the analysis is available at https://github.com/andrewkjar/SynNotch_scRNAseq. Single-cell RNA sequencing data generated in this study are available in ArrayExpress under accession number E-MTAB-15075.

### Statistical Analysis

Statistical analysis was performed in GraphPad Prism version 10.2.2. Normality was determined using Shapiro-Wilk tests before performing F-tests to probe for either equal or unequal variance. Each GFP MFI sender cell bar graph displays the average of each of three experiments, with every experiment consisting of biological triplicates, and include error bars indicating standard deviation (SD). SynNotch reporter activation studies display the average of five biological replicates from one experiment and include error bars indicating SD. ImageJ analysis from floor plate experiments includes the average of five fields of view each for two biological duplicates per experiment over three total experiments. Captions note specific tests performed on all data after checking for normality and variance distribution, generally as follows: for pairwise comparisons, if samples passed the normality test, a parametric test was run (Student’s *t* test for equal variance; Welch’s *t* test for unequal variances). In cases where sample distributions were not normal, a Mann–Whitney test was performed to compare ranks. Multiple comparisons were performed with a one-way ANOVA. If data were found to have unequal variances, a Welch’s ANOVA test was performed. If the data were found to be not normal, a Kruskal–Wallis ANOVA test was performed. Tukey’s multiple comparison was used as a post hoc test. If needed, an outlier assessment was performed before moving forward with the appropriate statistical test.

## Results

### PDGFRβ_TM_ GFP-Ligand Exhibits Deficiencies in synNotch hPSC Activation Studies

SynNotch has been used as a tool to induce user-defined transcriptional outputs, either through juxtacrine signaling or by way of ligand-presenting biomaterials.^21,22,25^ Both the extracellular and intracellular domains can be replaced in synNotch receiver cells, allowing for vast customization of input-to-output assignments. Upon recognition of a target molecule on a neighboring cell (sender cell) or immobilized surface, the intracellular domain within synNotch receiver cells is cleaved from the conserved Notch domain, which occurs through proteolysis via a disintegrin and metalloproteinase 10 and the gamma-secretase complex,^43^ allowing for highly localized transgene expression in the receptor-expressing receiver cell^21^ (Figure 1A).

The typical design for the synNotch ligand presented by sender cells consists of a target ligand (e.g., GFP) mounted on PDGFRβ_TM_, a truncated form of the PDGFRβ transmembrane domain, in synNotch sender cells (Figure 1A). This PDGFRβ_TM_ GFP-ligand has rendered robust synNotch activation in a variety of cell types, including HEK293s,^21,26,27^ T cells,^21,44−47^ L929 fibroblasts,^21−25^ and mouse ESCs.^28^ With this in mind, we generated an SB transposon vector constitutively expressing a GFP-ligand on the PDGFRβ_TM_ domain and transfected H9 hESCs to create synNotch sender cells (PDGFRβ_TM_ H9s). However, upon performing flow cytometry, we observed that PDGFRβ_TM_ H9s lacked any GFP expression, even in an antibiotic-selected population (Figure 1B,C, Supplemental Figure 1C). Unsurprisingly, when we cocultured these GFP-null PDGFRβ_TM_ H9s with synNotch receiver H9s engineered to express mCherry upon activation (mCherry H9s), we saw no evidence of synNotch activation (Figure 1D,E, Supplemental Figure 1F,G).

Surprised by the absence of GFP-ligand expression in PDGFRβ_TM_ H9s, we generated K562 and HEK293 GFP-ligand cell lines using the same SB vectors deployed in the PDGFRβ_TM_ H9s hESCs (PDGFRβ_TM_ K562s and PDGFRβ_TM_ HEK293s). K562 and HEK293 cells have been previously productively used as PDGFRβ_TM_ senders in numerous publications evaluating activation of non-hESCs.^21,26,44^ Compared to PDGFRβ_TM_ HEK293s and PDGFRβ_TM_ K562s, PDGFRβ_TM_ H9s showed lower GFP expression via flow cytometry (Figure 1B,C). PDGFRβ_TM_ HEK293s exhibited a ∼27-fold increase in GFP fluorescence intensity, and PDGFRβ_TM_ K562s displayed ∼23-fold increase, compared to their respective WT controls (Supplemental Figure 1A,B). Anti-GFP immunolabeling in fixed but unpermeabilized cells also showed little to no GFP positivity in PDGFRβ_TM_ H9s compared to PDGFRβ_TM_ HEK293s (Supplemental Figure 2), indicative of low levels of surface GFP presentation in PDGFRβ_TM_ H9s.

These results with HEK293s and K562s confirm that the lack of GFP expression in H9s is not due to the vector design. Thus, in a follow-up study, we cocultured either PDGFRβ_TM_ K562s or PDGFRβ_TM_ HEK293s with synNotch-mCherry H9s. After 72 h, cocultures with PDGFRβ_TM_ K562s or PDGFR_TM_ HEK293s exhibited ∼ 12-fold or ∼ 48-fold enhancement of anti-mCherry immunolabeled intensity, respectively, compared to WT cocultures lacking a GFP-ligand input (Supplemental Figure 1D–F). PDGFRβ_TM_ K562s generated ∼12% mCherry+ cells, while PDGFRβ_TM_ HEK293s induced ∼ 28% mCherry+ cells (Figure 1D). Because only ∼50% of cells within the coculture are synNotch receivers, we can estimate synNotch activation by doubling the percentage of mCherry+ cells. Thus, PDGFRβ_TM_ K562s are capable of inducing activation of ∼25% synNotch-H9 receiver cells while PDGFRβ_TM_ HEK293s can induce activation of ∼55% receivers. We therefore concluded that the lack of synNotch activation in H9 hESC cocultures was attributable to a deficiency of the PDGFRβ_TM_ chassis for presenting GFP in the context of an H9 hESC juxtacrine signaling platform. We thus began to investigate alternative transmembrane motifs to allow for synNotch activation in hESC:hESC cocultures.

### Alternative Transmembrane Motifs Allow for Robust synNotch Activation in hESC Cocultures

Given the lack of GFP expression and mCherry activation with PDGFRβ_TM_ H9s, we investigated an alternative transmembrane protein that could potentially enhance GFP-ligand expression and subsequent synNotch activation in H9 hESC synNotch cocultures. H9s were engineered with vectors encoding a ligand composed of the transmembrane and intracellular domains of E-Cadherin displaying GFP on the extracellular surface^31^ (Ecad H9s). In addition to being highly expressed in WT hPSCs,^48,49^ the intracellular domain of E-Cadherin also interfaces with the actin cytoskeleton of cells, suggesting an advantage for stably presenting synNotch ligand and providing the requisite ∼ 12 pN force for receiver cell activation.^50,51^ Further, GFP mounted upon the E-Cadherin transmembrane and extracellular domains has been previously applied to drive synthetic multicellular assembly in conjunction with a GFP-binding partner also tethered to the E-Cadherin chassis,^31^ establishing the utility of E-Cadherin in mediating potent cell–cell interactions via orthogonal presentation of extracellular motifs.

**Figure 2:**
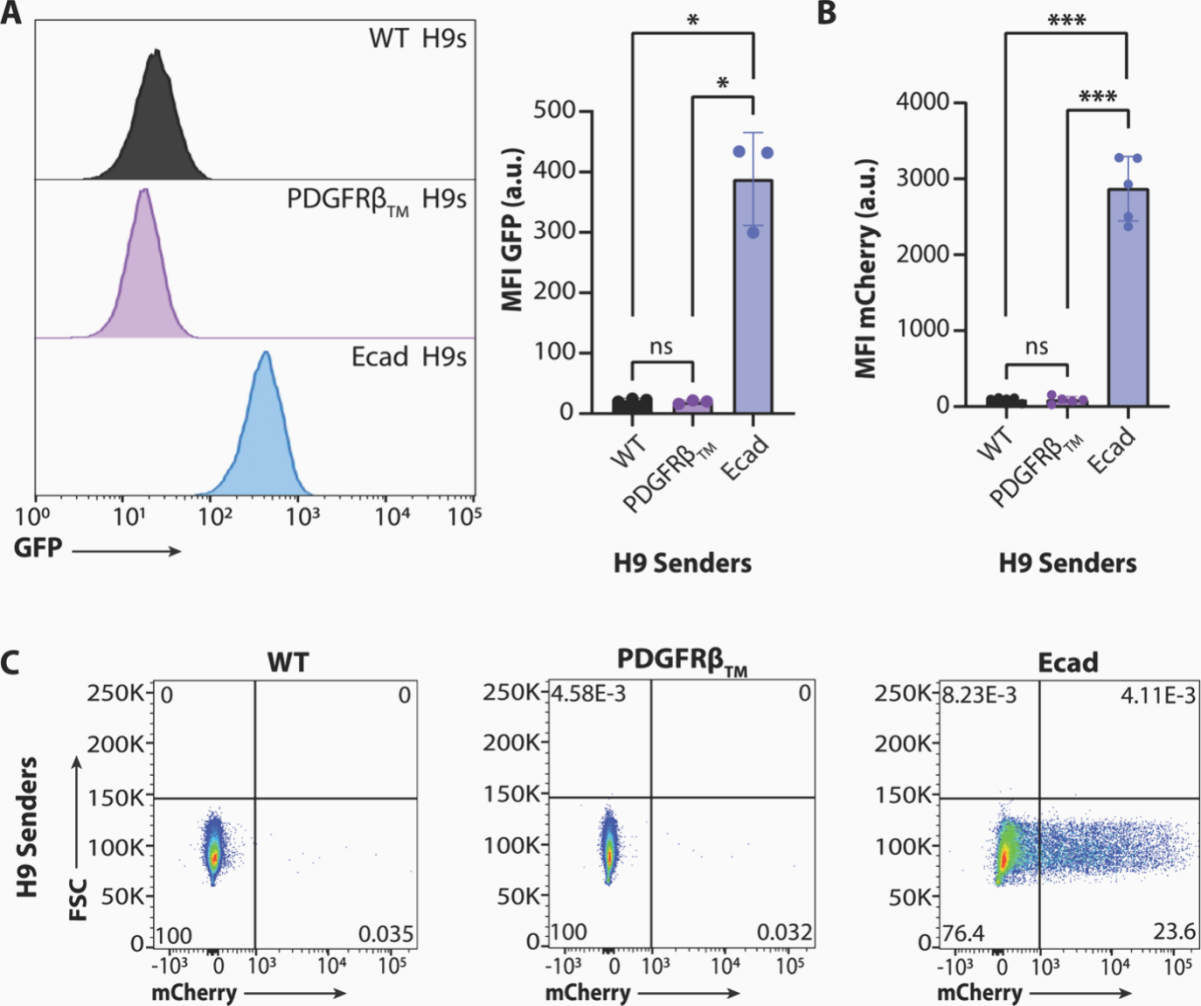
E-Cadherin serves as a productive GFP chassis for H9 senders. (A) Flow cytometry results of PDGFRβ_TM_-mounted GFP versus E-Cadherin-mounted GFP in H9 senders. Welch’s ANOVA with Tukey’s multiple comparisons post hoc: ns: not statistically significant, *p < 0.05. (B,C) Flow cytometry results of cocultures composed of either WT, PDGFRβ_TM_, or Ecad H9 senders and synNotch-mCherry H9 receivers at a 1:1 ratio. Welch’s ANOVA with Tukey’s multiple comparisons post hoc: ns: not statistically significant, ***p < 0.001.

Compared to PDGFRβ_TM_ H9s, Ecad H9s showed significantly higher GFP expression (Figure 2A). Fixed and unpermeabilized Ecad H9s immunolabeled with an anti-GFP antibody showed higher extracellular GFP localization compared to PDGFRβ_TM_ H9s (Supplemental Figure 2). Ecad H9s were cocultured with mCherry H9s, and anti-mCherry immunolabeled expression indicative of synNotch activation was measured via flow cytometry. Ecad H9 cocultures induced significant activation (Figure 2B,C, Supplemental Figure 1F,G) of ∼47% synNotch receiver cells (Figure 2C). In addition to E-Cadherin, we also considered DARC, a silent chemokine receptor found on erythrocytes, which has been used to present engineered surface T cell engagers for cancer immunotherapies.^32^ While Ecad H9s outperformed DARC in terms of receiver cell activation (Supplemental Figure 1F), DARC H9s displayed robust levels of overall GFP and surface expression (Supplemental Figure 1C, Supplemental Figure 2). These results highlight E-Cadherin tethered-GFP-ligand as a functional alternative to the earlier PDGFRβ_TM_ variant.

To generalize these findings in H9 hESCs, we also engineered KOLF2.1J hiPSCs as GFP-ligand sender cells (i.e., PDGFRβ_TM_ KOLF2.1Js and Ecad KOLF2.1Js). Distinct from our observations in H9 cells, GFP expression was relatively comparable between PDGFRβ_TM_ KOLF2.1Js and Ecad KOLF2.1Js (Supplemental Figure 3A). However, when in coculture with synNotch receiver KOLF2.1Js engineered to express mCherry upon activation (mCherry KOLF2.1Js), Ecad KOLF2.1J senders induced a significantly higher fraction of mCherry- expressing cells (∼100%) compared to only modest activation induced by PDGFRβ_TM_ KOLF2.1J senders (∼10%) (Supplemental Figure 3B–D), further suggesting that the PDGFRβ_TM_ GFP-ligand exhibits a deficiency in surface trafficking or membrane stability in hPSCs. Based on these results with two synNotch-hPSC lines showing substantial improvements over PDGFRβ_TM_, subsequent experiments were performed using GFP presented on E-Cadherin as the synNotch ligand.

### SynNotch hESC Cocultures Can Generate Markers of an Early Floor Plate-like Phenotype

To understand their use in modeling morphogenetic processes and generating a floor plate-like fate, receiver H9s were engineered with synNotch-driven SHH (SHH H9s). To interrogate whether SHH H9s can induce a floor plate-like fate, SHH H9s were cultured with magnetic beads coated with anti-c-Myc antibody (anti-c-Myc beads), which can recognize a c-Myc-epitope tag. Because the synNotch receptor in the SHH H9 receivers is N-terminally tagged with a c-Myc epitope, anti-c-Myc beads can activate synNotch-driven SHH expression (Supplemental Figure 4A). SHH H9s were seeded with anti-c-Myc beads ((+)Myc) in neural induction medium to encourage neural conversion.^52^ The (+)Myc cultures were compared to two controls: untreated cultures or cultures treated with beads coated in antihemagglutinin (HA) antibody (anti-HA beads, (+)HA). After 5 days in culture, the hallmark floor plate marker, *FOXA2*(13,53,54) was substantially elevated in SHH H9 (+)Myc cultures (Supplemental Figure 4B,C). Both endogenous *SHH* and transgene *SHH* expression increase throughout the 5 days in culture (Supplemental Figure 4D–H). The observed increase in endogenous *SHH* expression is reflective of the signaling center function of floor plate cells.

**Figure 3:**
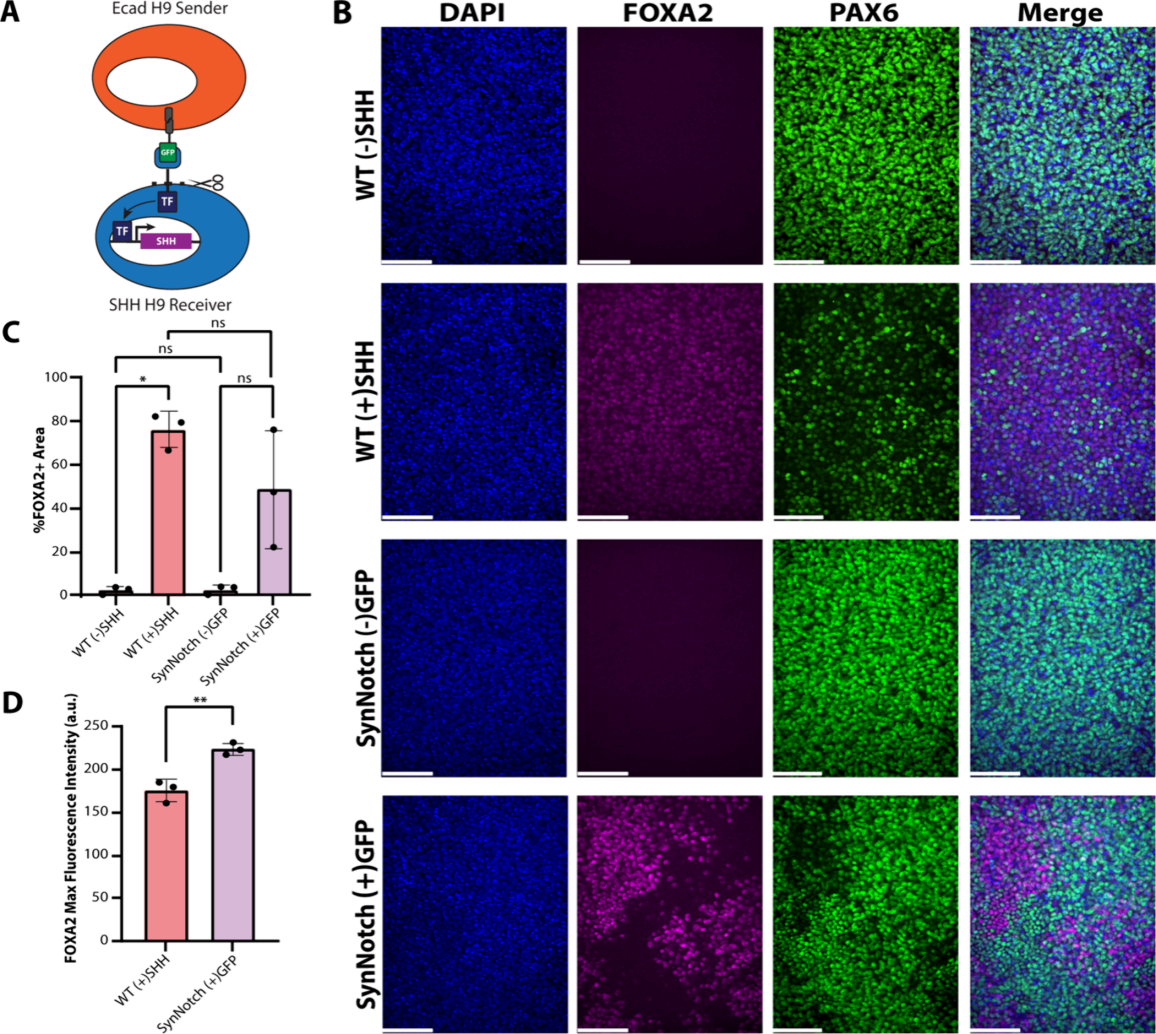
SynNotch-driven SHH leads to an increase in the floor plate marker FOXA2 in cells with correspondingly low PAX6 expression. (A) Ecad H9 senders mounted with GFP were combined with SHH H9 synNotch receivers. Following cleavage at the transmembrane core and translocation of a transcription factor (TF) to the nucleus, SHH H9 receivers express the morphogen SHH. (B) H9s were cultured in neural induction medium for 11 days before fixing cells and performing immunofluorescence. WT H9s were either cultured without recombinant SHH (WT (−)SHH) or with recombinant SHH protein (WT (+)SHH). SynNotch SHH H9s were placed in a 1:1 coculture with either WT H9s (SynNotch (−)GFP) or GFP-presenting Ecad H9s (SynNotch (+)GFP). Scale bar: 100 μm. (C) DAPI+ cells were assessed for %FOXA2 positivity from each field of view. Welch’s ANOVA with Tukey’s multiple comparisons post hoc: ns: not statistically significant, *p < 0.05. (D) The maximum fluorescence intensity of FOXA2+ cells was calculated for groups containing SHH. Student’s *t* test: **p < 0.01.

After confirming SHH signaling in activated SHH H9s, Ecad H9s were combined with SHH H9s at a 1:1 ratio (Figure 3A) and seeded in neural induction medium. This combination of Ecad H9s and SHH H9s in coculture (SynNotch (+)GFP) was compared against WT H9s in neural induction medium supplemented with recombinant SHH protein (WT (+)SHH). In two negative control groups, SHH H9s were combined in coculture with WT H9s (SynNotch (−)GFP) or WT H9s were cultured without recombinant SHH protein (WT (−)SHH). As expected, absence of SHH, as in either the WT (−)SHH or SynNotch (−)GFP groups, resulted in cultures lacking FOXA2 immunofluorescence and instead showed high levels of PAX6,^13,53^ a neuroectodermal/dorsal progenitor marker that decreases in expression upon exposure to SHH (Figure 3B). Thus, this population of cells maintained neural progenitor cell specification, lacking any additional morphogenetic patterning. Distinctly, WT (+)SHH cultures showed uniformly distributed FOXA2 expression concurrent with a reduction in PAX6 levels (Figure 3B). SynNotch (+)GFP cultures also showed regions of high FOXA2 signal corresponding with low PAX6 signal (Figure 3B), highlighting the dorsal-ventral division seen during innate neural tube patterning. Interestingly, SynNotch (+)GFP cultures showed higher levels of heterogeneity and fewer FOXA2+ cells compared to WT (+)SHH cultures (Figure 3B). However, FOXA2 signal in the SynNotch (+)GFP group exhibited higher maximum fluorescence intensity values compared to WT (+)SHH cultures (Figure 3D). Given the short signaling range of SHH (50–100 μm),^55,56^ this suggests that synNotch-driven SHH production exerted locally potent effects on cell fate specification. We also speculated that the reduced number of FOXA2+ cells in SynNotch (+)GFP cultures is likely due to an estimated 50% of receiver cells being activated, based on prior flow cytometry experiments showing only ∼47% of synNotch-H9s were activated via Ecad H9s in coculture (Figure 2C). Nevertheless, these results reveal that synNotch-driven SHH production induced by Ecad-GFP senders is competent to produce FOXA2+ progenitors from hPSCs.

**Figure 4:**
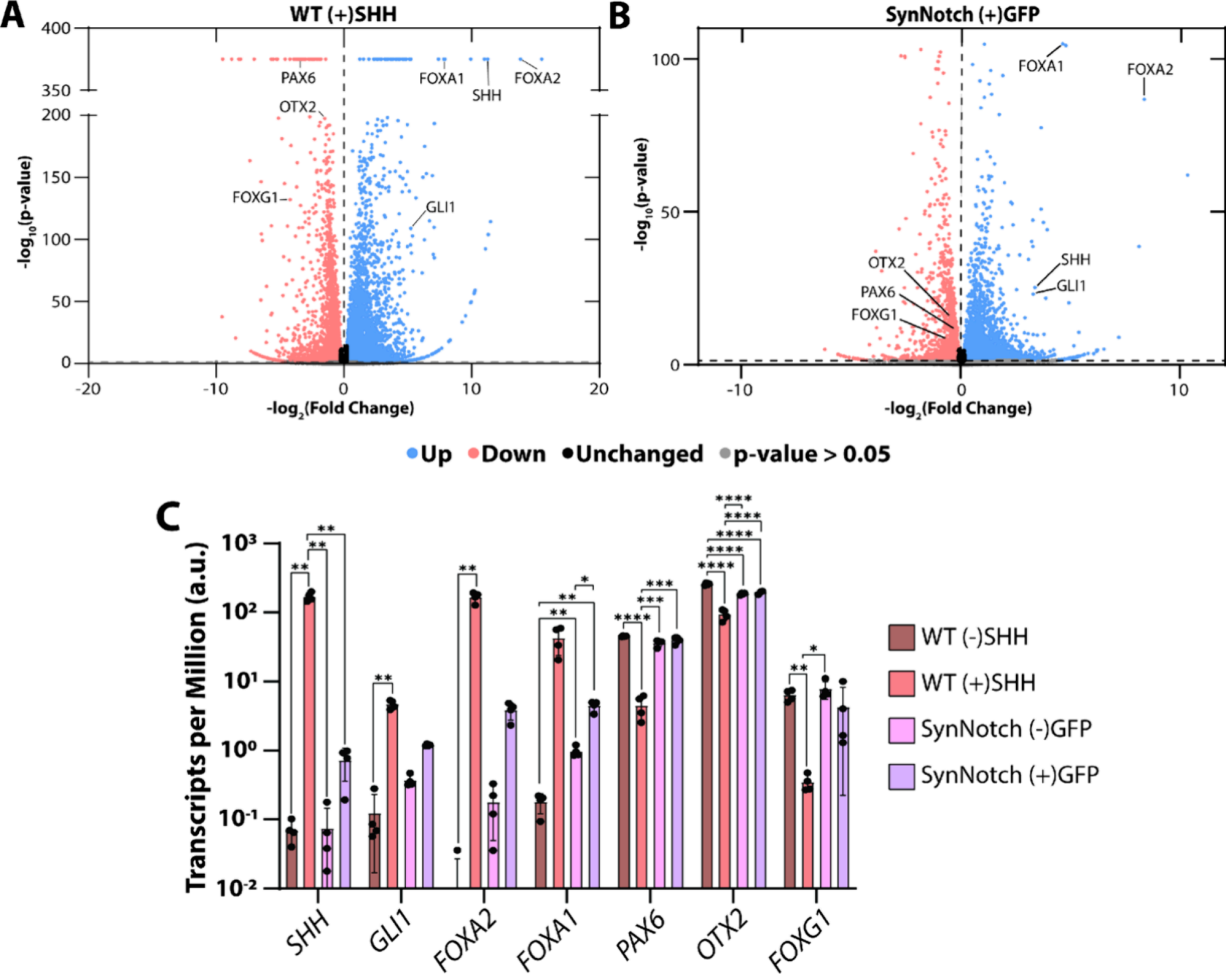
Gene expression analysis of bulk RNA sequencing data. Volcano plots of fold change in gene expression plotted against statistical significance for (A) WT H9s with recombinant SHH protein (WT (+)SHH) and (B) SHH H9 receivers cocultured with GFP-Ecad H9s (SynNotch (+)GFP). Both volcano plots are compared to the WT H9s without recombinant SHH protein condition (WT (−)SHH). Labeled genes are either SHH-responsive (SHH and *GLI1*), floor plate-relevant (*FOXA2* and *FOXA1*), or dorsal-anterior-relevant (*PAX6*, *OTX2*, and *FOXG1*). (C) Bar graph of average TPM values for genes labeled in (A,B), illustrating expression levels across all four conditions (WT (−)SHH, WT(+)SHH, SynNotch (−)GFP, and SynNotch (+)GFP). Genes are categorized as SHH-responsive, floor plate-relevant, or dorsal-anterior-relevant. *SHH*, *FOXA1*, *PAX6*, *FOXG1*: Welch’s ANOVA with Tukey’s multiple comparisons post hoc; *GLI1*, *FOXA2*: Kruskal–Wallis ANOVA with Tukey’s multiple comparisons post hoc; *OTX2*: one-way ANOVA with Tukey’s multiple comparisons post hoc; *p < 0.05, **p < 0.01, ***p < 0.001, ****p < 0.001.

To further characterize the gene expression profiles of these populations, we performed bulk RNA sequencing and compared the two conditions with SHH (WT (+)SHH and SynNotch (+)GFP) against the WT (−)SHH condition. When comparing differentially expressed genes, both WT (+)SHH cultures and SynNotch (+)GFP cultures exhibited an increase in Hallmark Hedgehog Signaling^57^ genes such as *GLI1* and *SHH* (Figure 4A,B). Both conditions also generated an increase in floor plate markers *FOXA1* and *FOXA2.*(58−60) We also saw a reduction in the dorsal marker *PAX6* along with *OTX2* and *FOXG1*, two forebrain markers, in both of these conditions (Figure 4A,B). Taken together, these data indicate both the traditional differentiation strategy and synNotch-driven differentiation render an expected increase in SHH-dependent genes. They also show these conditions adopting a more ventral identity due to SHH, which suppresses dorsal-anterior fates associated with the forebrain. However, SynNotch (+)GFP cultures tended to have lower fold induction compared to WT (+)SHH cultures, consistent with protein-level, immunofluorescence results. To compare expression levels more broadly across conditions, we visualized the average transcripts per million (TPM) values of SHH-responsive, floor plate, and dorsal-anterior genes across all four populations (Figure 4C). This analysis confirmed overall elevated expression of SHH targets and floor plate markers in WT (+)SHH cultures, along with reduced expression of dorsal-anterior genes in both WT (−)SHH and SynNotch (−)GFP cultures. In contrast, SynNotch (+)GFP cultures exhibited generally lower TPM values than WT (+)SHH cultures, with only a modest increase in the floor plate markers *FOXA1* and *FOXA2*. This likely reflects the heterogeneous nature of the SynNotch condition, in which only a subset of the coculture adopts a floor plate-like fate— which aligns with the immunofluorescence data. With this in mind, we also performed single-cell RNA sequencing to further analyze the floor plate-like cells derived from this heterogeneous SynNotch (+)GFP condition.

### Molecular Analysis Shows Emergence of Floor Plate-like Fates

**Figure 5:**
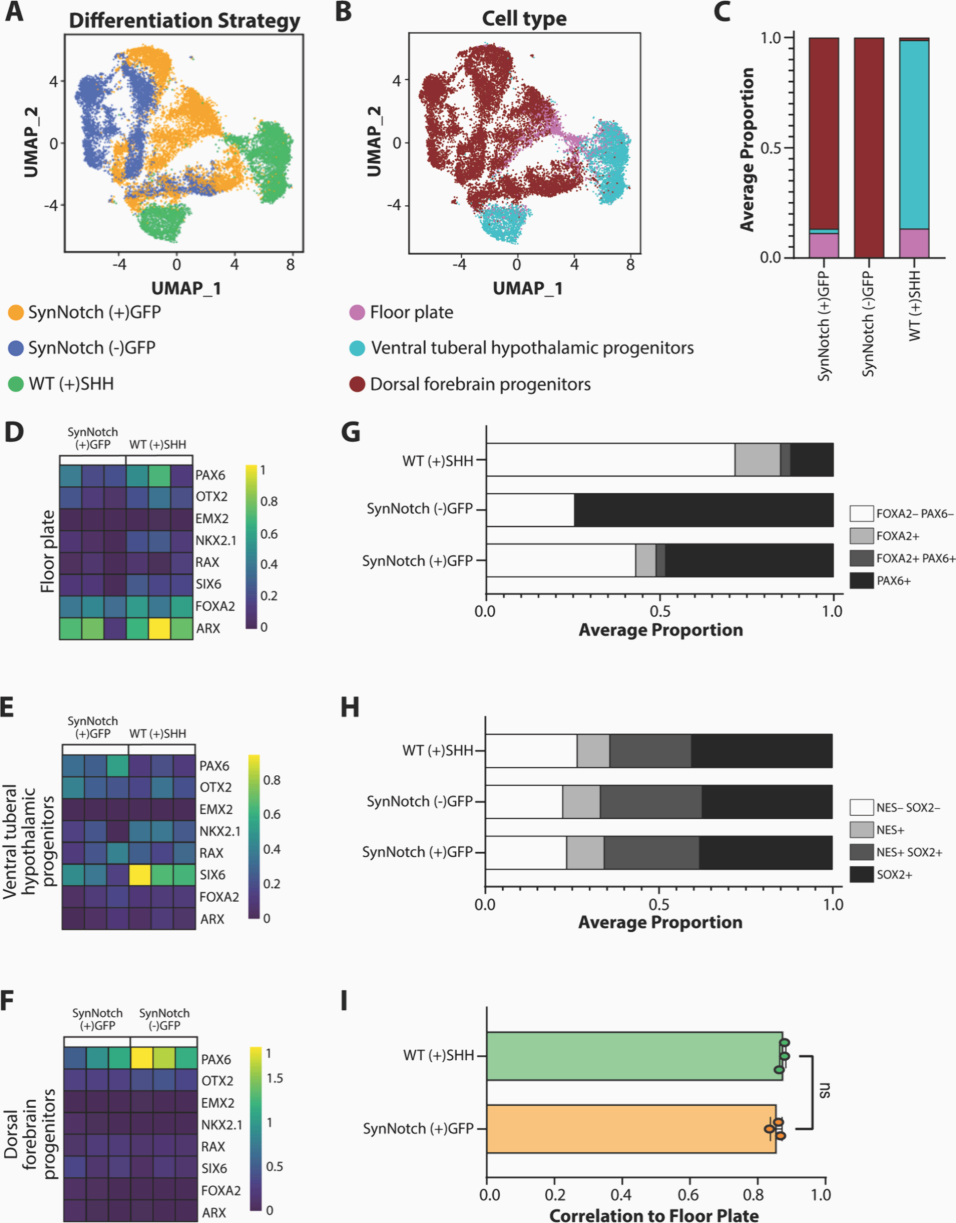
Annotation of cells analyzed for single-cell RNA sequencing. (A) UMAP of integrated cells, colored by annotated cell type. (B) UMAP colored by differentiation strategy. (C) Average proportion of cell types from each differentiation strategy. Average from *n* = 3 differentiations each. (D–F) Heatmaps of key marker genes for each cell population, across *n* = 3 biological samples each. Averaged expression levels per cell type: floor plate (D), ventral tuberal hypothalamic progenitors (E), and dorsal forebrain progenitors (F). (G) Average proportion of cell types annotated by *FOXA2* and *PAX6* expression from each differentiation method. Average from *n* = 3 differentiations each. (H) Average proportion of cell types annotated by *NES* and *SOX2* expression from each differentiation method. Average from *n* = 3 differentiations each. (I) Correlation to floor plate cells from 4–6 week human embryos.^42^ Student’s *t* test, ns: not statistically significant.

Single-cell RNA sequencing was performed to compare WT (+)SHH, SynNotch (−)GFP, and SynNotch (+)GFP cultures. Cells passing QC (Supplemental Figure 5A) were visualized using UMAP and annotated based on canonical markers (Figure 5A–C, Supplemental Figure 5B–E). Critically, both WT (+)SHH and SynNotch (+)GFP conditions yielded floor plate progenitors (*FOXA2*, *ARX*), while the SynNotch (−)GFP condition did not (Figure 5B,C). When separated based on cell annotation identity, floor plate-like cells showed similar expression levels of *FOXA2*/*ARX* regardless of differentiation strategy (adjusted *p*-value >0.05 for all markers) (Figure 5D). However, both differentiation strategies gave rise to unique alternative fates (Figure 5E–H). Whereas the WT (+)SHH culture consisted mostly of ventral tuberal hypothalamic progenitors (*NKX2.1, RAX, SIX6*), SynNotch (+)GFP cultures yielded a high proportion of dorsal forebrain progenitors (*PAX6, OTX2*) (Figure 5B,C). Consistent with the immunofluorescence data (Figure 3), WT (+)SHH cultures generated fewer *PAX6* cells and a greater number of *FOXA2* cells compared to SynNotch (+)GFP cultures. Lastly, all differentiation strategies produced large portions of *SOX2*/*NESTIN*-expressing cells (Figure 5H, Supplemental Figure 5G), indicating cellular commitment to neuroectoderm and fates of the CNS. Despite the pronounced differences in overall cellular composition between WT (+)SHH and SynNotch (+)GFP cultures, the floor plate progenitors generated under both conditions exhibited highly similar marker expression profiles (Figure 5D), suggesting that synNotch-mediated SHH induction is sufficient to specify floor plate identity in a manner comparable to exogenous SHH treatment.

To contextualize the floor plate-like molecular profiles, we sought to benchmark cell phenotypes to in vivo transcriptomic data sets. We compared these data to the Brainspan Atlas,^41^ which transcriptionally mapped human brain development from embryogenesis through adulthood. We found that floor plate-like cells derived from either WT (+)SHH or SynNotch (+)GFP conditions mapped similarly (Supplemental Figure 6A), showing correlation to the dorsal thalamus or mediodorsal nucleus of the thalamus. Comparison to primary developmental human floor plate tissue derived from 4 to 6 week old embryos^42^ showed similar correlation levels from cells derived from either differentiation strategy containing SHH (Figure 5I, Supplemental Figure 6B). Only a few differentially expressed genes were detected upon direct comparison of the floor plate-like cells from each method providing SHH, again underscoring the molecular similarity of the floor plate progenitors derived from each condition (Supplemental Figure 6C,D).

Building on these findings, we further investigated how varying sender:receiver ratios influenced synNotch activation and hPSC differentiation. In a follow-up study, we compared sender:receiver ratios of 3:1, 1:1, and 1:3 for mCherry activation efficiency and FOXA2 immunofluorescence. As expected, altering the ratio impacted signaling outcomes. Co-culturing Ecad H9 senders with either mCherry H9 or SHH H9 receivers at a 3:1 ratio resulted in increased synNotch activation compared to the standard 1:1 ratio or even 1:3 conditions (Supplemental Figure 7A,B). Furthermore, the 3:1 Ecad H9 sender:SHH H9 receiver ratio yielded the highest FOXA2+ area; however, the 3:1 cultures exhibited lower maximum fluorescence intensity than 1:1 cocultures (Supplemental Figure 7C), consistent with trends observed in Figure 3D. These findings suggest that tuning sender:receiver ratios can modulate synNotch-driven floor plate progenitor specification. Taken together, our results illustrate that Ecad H9 senders dramatically influence floor plate-like specification by SHH H9s and that outcomes can be modulated by varying sender:receiver ratios.

## Discussion

The synNotch platform has been widely used as a juxtacrine signaling channel in numerous synthetic morphogenesis applications that involve patterning of immortalized fibroblasts and epithelial cells or differentiation of mouse ESCs, but few studies have deployed synNotch to regulate functions of hPSCs—cells that are of great interest in the field due to their capacity to give rise to any cell type in the developing embryo. While prior works have demonstrated success in using synNotch to regulate hPSC behaviors in response to ligand-presenting biomaterials, this report represents, to our knowledge, the first to demonstrate synNotch signal propagation in an hPSC juxtacrine sender:receiver configuration. To accomplish this, we first had to overcome an apparent incompatibility of the transmembrane PDGFRβ_TM_ GFP-ligand with use in an hPSC:hPSC coculture system, which was observed in both H9 hESCs and KOLF2.1J hiPSCs. HEK293 and K562 cell lines overexpressing PDGFRβ_TM_ GFP-ligand were competent to activate synNotch-hPSCs, indicating that the inability of PDGFRβ_TM_ hPSCs to productively stimulate synNotch-hPSCs is not due to an intrinsic deficiency of PDGFRβ_TM_-based ligand presentation to hPSCs. Therefore, our results demonstrate that hPSCs fail to adequately activate synNotch hPSC receivers via the PDGFRβ_TM_ GFP-ligand variant, posing a severe bottleneck in efforts to deploy synNotch in synthetic morphogenesis hPSC sender:hPSC receiver coculture experiments.

We found that alternative chassis for synNotch ligand, including either the transmembrane decoy receptor DARC or the E-Cadherin transmembrane and intracellular domains, enable adequate GFP-ligand presentation in hPSCs to then stimulate potent synNotch-driven responses in receiver hPSCs. Finally, we show the ability of hPSC synNotch sender:receiver cocultures to drive the emergence of floor plate-like progenitor cells on the basis of synNotch-regulated SHH production. Collectively, our results provide a means to implement synNotch juxtacrine signaling efficiently in hPSC systems and motivate widespread adoption of alternative ligand types in artificial developmental systems that make use of hPSCs and the synNotch signaling channel.

Interestingly, we do not observe a consistent or universal ligand chassis that performs best across HEK293s, K562s, and hPSCs: though E-Cadherin functions highly in the context of hPSC senders to activate synNotch-hPSCs, Ecad K562 sender cells only negligibly activate synNotch-hPSCs. The minimal activation induced by Ecad K562 senders contrasts starkly with the potent induction achieved by PDGFRβ_TM_ K562 senders (Supplemental Figure 1E), despite similar levels of ligand expression between the two sender lines (Supplemental Figure 1B). Similar results were attained with KOLF2.1J hiPSCs, where overall expression of PDGFRβ_TM_- and Ecad-based ligands were similar but major differences in their ability to induce synNotch activation in receiver cells was observed. A potential explanation for this result is that these ligand variants have differential capacity to traffic to and stably integrate into the membranes of sender cells. These results highlight that efficient juxtacrine signaling via the synNotch platform entails optimization of ligand chassis in a context- and cell type-dependent manner.

Our attempts to leverage synNotch to derive ventral floor plate-like progenitors provides several insights. First, our results indicate that synNotch ligand is required to induce meaningful differentiation in SHH-engineered receiver cells, suggesting that basal levels of synNotch-regulated payload transgenes have minimal impact on hPSC phenotype, consistent with prior reports.^20,22,44^ Additionally, the juxtacrine sender:receiver differentiation scheme deployed here resulted in a diverse cell population. Several elegant studies have demonstrated that the signaling range of SHH is on the order of 50–100 μm (∼5 cell diameters)^55,56^ and is influenced by post-translational lipid modification of SHH, SHH interactions with extracellular matrix components in the cellular microenvironment, and the relative abundance of Patched and Hedgehog-interacting protein in cells within the niche.^56,61,62^ In light of the prospect of having activated ∼50% of H9 receivers to express SHH transgene, high levels of variability were expected. We were, however, struck by the relative abundance of PAX6+ cells. Considering the high levels of FOXA2 expression observed in our cultures, the predominance of PAX6+, dorsal-like populations suggests that further optimization of the sender:receiver ratio may enhance both the fraction of synNotch-activated cells and outcomes of synNotch-hPSC differentiation schemes, as indicated by our follow-up study varying sender:receiver ratios. Further studies need to be performed to fully uncover the sender:receiver synNotch ratio that is optimal for this system as well as other synNotch juxtacrine applications. In a prior report, De Santis et al.^13^ regulated SHH expression in hPSCs via an optogenetic platform and, like us, observed varied expression of markers of ventral progenitor specification, even in regions of optical illumination that renders irreversibly constitutive SHH production. The level of heterogeneity observed in such an experiment arose despite the presence of a more uniform initial hPSC population: all cells in the optogenetic experiments were competent to respond to light input by expressing SHH, rather than having a mixed configuration of only 50% cells activated to produce SHH as in our cocultures. Thus, these results align with our findings that a uniform ventral floor plate population may not emerge from an hPSC differentiation that involves biomimetic signaling mechanisms, and artificially regulated production of morphogens may offer a means to faithfully reflect outcomes of in vivo differentiation paradigms, which rarely give rise to homogeneous cell populations.

Important to note, there is also a difference in synNotch-driven SHH and that of the recombinant version of the protein used in our studies. While synNotch-driven SHH produced by SHH H9s is a codon-optimized version of human SHH, the recombinant SHH we used was based on prior studies attempting to derive floor plate from hPSCs. This recombinant form of SHH, known as C25II SHH, is a mutant form of mouse SHH with an N-terminal modification that involves substitution of cysteine with isoleucine residues, allowing for enhanced signaling potency.^61,63,64^ While we sought to compare our results against a methodology commonly used to differentiate hPSCs toward floor plate-like cells,^35^ the primary objective of our differentiation strategy focused on cell-directed patterning by a biomimetic system that encourages these developmental processes. Therefore, we opted to engineer SHH H9s with a codon-optimized version of the full-length human SHH protein instead of this enhanced, modified SHH protein. In taking this approach, our studies may not have created the most favorable comparative condition for synNotch-SHH cells. Nonetheless, the synNotch platform proved competent to drive emergence of developmentally relevant CNS cells.

Synthetic gene circuits, such as doxycycline-inducible^12^ and optogenetically^17^ driven expression of SHH, have been used in innovative brain organoid studies to investigate how a polarized SHH source influences topographical specification of CNS cell subtypes in the organoid. In these studies, investigators need to stimulate SHH production for a specified period. Distinct from these platforms, one attractive feature of synNotch is that it endows cells with the capacity to interpret the spatial contents of their microenvironment, giving them the power to discern how to respond to emergent properties of cellular collectives. Given the roadmap provided here for hPSC sender:receiver synNotch studies, future studies are poised to deploy this signaling modality in combination with reporters that regulate expression of ligands such as E-Cadherin in response to cell fate changes within organoids. Foreshadowing this, our earlier work showed that synNotch signals can be propagated based on expression of GFP-ligand from native loci such as the *PAX6* locus,^26^ which serves as a transient, early marker of a transition from pluripotency toward a neuroectodermal progenitor upstream of ventral floor plate specification. Combined with work presented here, such advances open several avenues to determine the design rules required to elaborate the hierarchical, sequentially derived structures relevant to CNS patterning in a manner that relies on cell:cell interactions for templating instructions rather than user-specified stimulation via doxycycline or optical inputs.

While this work is limited to an early floor plate-like fate, additional patterning factors, such as the WNT pathway activator CHIR99021 and fibroblast growth factor (FGF), can specify advanced floor plate identities, including anterior and posterior floor plate fates, respectively. While we elected here to use synNotch to drive only expression of SHH, the modular and orthogonal nature of synNotch enables incorporation of multiple unique synNotch receptors and corresponding payload transgene circuits into a single cell.^21,24,25^ Future studies could explore using such receiver cells to conditionally regulate WNT or FGF signaling to further pattern hPSCs toward lineages of interest.

The bulk of our studies here were restricted to the widely used hESC H9 line. We did confirm similar outcomes in the KOLF2.1J hiPSC line, where PDGFRβ_TM_ KOLF2.1J activation was significantly outperformed by Ecad KOLF.21Js when in coculture with mCherry KOLF2.1Js. While we anticipate that E-Cadherin will be more highly functional than PDGFRβ_TM_ in multiple other hPSC lines, our studies may inspire investigation to assess performance of various ligand configurations in user-selected stem cell lines prior to use. Finally, though we showed that E-Cadherin and DARC serve as productive ligands to activate juxtacrine synNotch signaling in hPSCs, unexplored ligand chassis may provide even more potent stimulus to drive synNotch responses. Future studies may uncover such improved ligand designs and would be welcomed to the expanding synthetic morphogenesis instrument set.

## Conclusions

In conclusion, we demonstrated that the typical synNotch ligand chassis, GFP mounted on PDGFRβ_TM_, failed to render potent synNotch activation in an hESC sender:receive coculture. We revealed that E-Cadherin and DARC represent viable alternative ligand configurations for use in hPSC:hPSC cocultures. We also demonstrated that levels of activation attained by an E-Cadherin-presented ligand were sufficiently potent to yield the emergence of floor plate-like progenitors in hESC-based synNotch cocultures. Our studies illustrate the value of attempting to guide cell fate specification via cell-mediated morphogenetic subroutines rather than by supraphysiologic stimulation by small molecule agonists/antagonists or recombinant factors. Considering the importance of juxtacrine signaling in development and the need to generate signaling reminiscent of developmental pathways in efforts to mimic development in a dish, our findings offer an expanded synNotch toolkit for use in synthetic morphogenesis studies leveraging hPSCs.

## Supporting Information Available

Additional experimental results, supporting microscopy images, and flow cytometry plots (PDF)

## Abbreviations

QC: quality control
PCA: principal component analysis
UMAP: uniform manifold approximation and projection
WT (−)SHH: WT H9s without recombinant SHH
WT (+)SHH: WT H9s with recombinant SHH
SynNotch (−)GFP: SHH H9s in coculture with WT H9s
SynNotch (+)GFP: SHH H9s in coculture with Ecad H9s
SD: standard deviation
TF: transcription factor
PDGFRβ_TM_ K562s: synNotch sender K562s with GFP-ligand on the PDGFRβ_TM_ domain
PDGFRβ_TM_ HEK293s: synNotch sender HEK293s with GFP-ligand on the PDGFRβ_TM_ domain
PDGFRβ_TM_ KOLF2.1Js: synNotch sender KOLF2.1Js with GFP-ligand on the PDGFRβ_TM_ domain
Ecad KOLF2.1Js: synNotch sender KOLF2.1Js with GFP-ligand on the extracellular surface of E-Cadherin
mCherry KOLF2.1Js: synNotch receiver KOLF2.1Js engineered to express mCherry upon activation
anti-c-Myc beads/(+)Myc: magnetic beads coated with anti-c-Myc antibody which can recognize a c-Myc-epitope tag
anti-HA beads/(+) HA: magnetic beads coated in antihemagglutinin antibody.
