# Supplementary material for: Induced Neural Progenitor Specification from Human Pluripotent Stem Cells by a Refined Synthetic Notch Platform
Source: ACS Synth Biol. 2025 May 6;14(5):1482–95. doi: 10.1021/acssynbio.4c00742 (PMC12090341; doi:10.1021/acssynbio.4c00742)
Supplement: Supplementary file 1 — sb4c00742_si_001.pdf [file sb4c00742_si_001.pdf]

Supporting Information

Induced neural progenitor specification from human pluripotent stem cells by a refined synthetic Notch platform

Catherine A. Hamann[1], Andrew Kjar[1], Hyosung Kim[2], Alan J. Simmons[3], Hannah J. Brien[1], Cheryl I. Quartey[4], Bonnie L. Walton[1], Ken S. Lau[3, 5, 6], Ethan S. Lippmann[2,5], and Jonathan M. Brunger*[1,5,6]

[1]Department of Biomedical Engineering, Vanderbilt University, Nashville, Tennessee, USA 37235, [2]Department of Chemical and Biomolecular Engineering, Vanderbilt University, Nashville, Tennessee, USA 37235, [3]Department of Cell and Developmental Biology, Vanderbilt University, Nashville, Tennessee, USA 37235, [4]Department of Biological Sciences, Vanderbilt University, Nashville, Tennessee, USA 37235, [5]Center for Stem Cell Biology, Vanderbilt University, Nashville, Tennessee, USA 37235 [6]Center for Computational Systems Biology, Vanderbilt University, Nashville, Tennessee, USA 37235

Supplemental Methods

KOLF2.1J hiPSC Culture Conditions. KOLF2.1J cells used in these studies were validated via short tandem repeat (STR) fingerprinting (ATCC) and tested negative for mycoplasma (Bulldog Bio 2523148). They were cultured in Stemflex (Gibco A3349401) on Geltrex-coated tissue culture plates. Once 70% confluent, cells were passaged using Accutase along with Stemflex supplemented with 10 μM Y-27632. A reverse transfection was performed as previously described with the SB transposase (Addgene 34879, a kind gift from Zsuzsanna Izsvak)[1] and the SB transposon encoding either the GFP-responsive LaG16-synNotch receptor or a variant of the GFP synNotch ligand. Cells were selected in 1.2 μg/mL puromycin for approximately 1 week before being sorted for GFP expression as previously described. Sender cells expressing a GFP-ligand either on the PDGFRβ transmembrane domain (GFP-PDGFRβ_{TM} KOLF2.1Js) or mounted in place of the ectodomain of E-Cadherin (GFP-Ecad KOLF2.1Js) were sorted such that they expressed the same levels of GFP. SynNotch receiver cells expressing mCherry (mCherry KOLF2.1Js) were sorted as described from the H9 receiver cells in the main text.

Antibody-labeled GFP Microscopy and Immunofluorescence. HEK293 and H9 hESC senders were fixed with 4% paraformaldehyde for 10 minutes, washed with PBS, and blocked with PBS+10% FBS. A rabbit anti-GFP primary antibody (Abcam, ab6556) was used at a 1:200 dilution and paired with a donkey anti-rabbit AlexaFluor 647 secondary (Invitrogen A-31573) at a 1:400 dilution. Cells were imaged using a Leica DMi8 epifluorescence microscope.

Flow Cytometry for KOLF2.1J mCherry readout of synNotch activation. At the second passage from thaw, KOLF2.1J hiPSC:KOLF2.1J hiPSC co-cultures were plated at a density of 400,000 cells/cm^2 in 96-well plates coated with Geltrex. Co-cultures were seeded at a 1:1 ratio of senders:receivers in Stemflex supplemented with 10 μM Y-27632. Twenty-four hours after

plating, medium was changed and replaced with mTeSR Plus excluding Y-27632. Seventy-two hours after plating, co-cultures were dissociated using Accutase before resuspending in PBS supplemented with 5% FBS. Inducible mCherry expression was examined using a BD LSRFortessa™ Cell Analyzer and analyzed using FlowJo.

SynNotch anti-c-Myc bead quantitative real-time PCR. At the second passage from thaw, H9 hESCs were plated at a density of 400,000 cells/cm^2 in 96-well plates coated with Geltrex. Magnetic beads coated with either anti-c-Myc antibody (anti-c-Myc beads) (Thermo Scientific 88842) or anti-hemagglutinin antibody (anti-HA beads) (Thermo Scientific 88836) were washed once with PBS by placing an Eppendorf tube containing the beads and PBS against a magnet. 4 uL of beads were used per 96-well. After approximately one minute when beads were pulled to one side of the Eppendorf, PBS was removed and the beads were resuspended in neural induction medium containing neurobasal medium (Gibco, 21103049) supplemented with 1% N2 supplement (Gibco, 17502048), 2% B-27 supplement without vitamin A (Gibco, 12587010), 1% GlutaMAX (Gibco, 35050061), 10 μM SB431542 (STEMCELL TECH, 72234), 100 nM LDN193189 (STEMCELL TECH, 72149), and 10 μM Y-27632. Medium was prepared (excluding Y-27632) and changed daily, and cells were taken out to 5 days in culture. 0.5 uL of beads were supplemented each day to account for any removal during media changes. Cells were lysed at days 1, 3, and 5 and mRNA was isolated using the PureLink RNA Mini Kit (Invitrogen 12183018A). mRNA was reversed transcribed into cDNA using SuperScript IV VILO Master Mix (Invitrogen 11766050) before performing quantitative real-time PCR (qRT-PCR) for gene expression using PowerTrack SYBR Green Master Mix (Applied Biosystems A46012). Samples were run on a Bio-Rad CFX96 with non-skirted low-profile plates (Thermo Scientific AB0700) with optical adhesive film (Applied Biosystems 4360954). Primer pairs used are listed in Supplementary Table 1. Relative

fold gene expression was calculated using the $\Delta\Delta C_t$ method using *GAPDH* as a reference gene. *FOXA2* and endogenous *SHH* were compared against their respective ΔC_t values at day 1. Transgene *SHH* was compared against SHH H9s in culture with anti-HA beads at each timepoint.

SynNotch Activation in Varied Sender:Receiver Ratios. At the second passage from thaw, mCherry H9 or SHH H9 receivers were seeded along with Ecad H9 senders, but in the following sender:receiver ratios: 3:1, 1:1, and 1:3. Co-cultures were prepared and maintained as previously described for 72 h (for mCherry readout) or 11 days (for FOXA2 readout). Cells were fixed with 4% paraformaldehyde for 10 minutes, washed with PBS, and permeabilized with permeabilization buffer (PBS containing 0.5% Triton-X100) for 10 minutes. After permeabilization, cells were blocked with blocking buffer (PBS containing 0.1% Triton-X100 and 5% FBS) prior to incubation with primary antibodies for one hour at room temperature. For mCherry immunolabeling, a rabbit anti-mCherry primary antibody [1:200; Cell Signaling 43590]) was used. For FOXA2 immunolabeling, a goat anti-FOXA2 primary antibody (1:200; R&D Systems AF2400) was used. Samples were then washed with PBS prior to application of secondary antibodies in blocking buffer (for mCherry detection: AlexaFluor 647 donkey anti-rabbit [1:400; Invitrogen A31573]; for FOXA2 detection: AlexaFluor 647 donkey anti-goat [1:400; Invitrogen A-21447]) for one hour at room temperature protected from light. Samples were then washed with PBS, counterstained with DAPI (1:1000; Thermo Scientific 62247), and washed with PBS a final time before being placed in blocking buffer. Samples were then imaged using a Leica DMi8 epifluorescence microscope. Images were analyzed using Fiji ImageJ and converted to 8-bit. To determine the percentage mCherry+ or FOXA2+ area, thresholding was performed for each channel. Area fraction was determined for each image. mCherry or FOXA2 signal was normalized to corresponding DAPI

images, where DAPI+ cells represented 100% area for each individual image. Maximum fluorescence intensity values were also determined for FOXA2+ area images.

Supplemental Table 1: Quantitative real-time PCR primer pairs for experiments relevant to Supplemental Figure 4.

GAPDH Forward Primer	ATGTTCGTCATGGGTGTGAA
GAPDH Reverse Primer	AGGGGTGCTAAGCAGTTGGT
FOXA2 Forward Primer	CCGACTGGAGCAGCTACTATG
FOXA2 Reverse Primer	TACGTGTTCATGCCGTTCAT
Endogenous *SHH* Forward Primer	CCGAGCGATTTAAGGAACTCACC
Endogenous *SHH* Reverse Primer	AGCGTTCAACTTGTCCTTACACC
Transgene *SHH* Forward Primer	CGCCTACGCTCCTCTTACAG
Transgene *SHH* Reverse Primer	TCAATCACGGCGTAGCAAGA

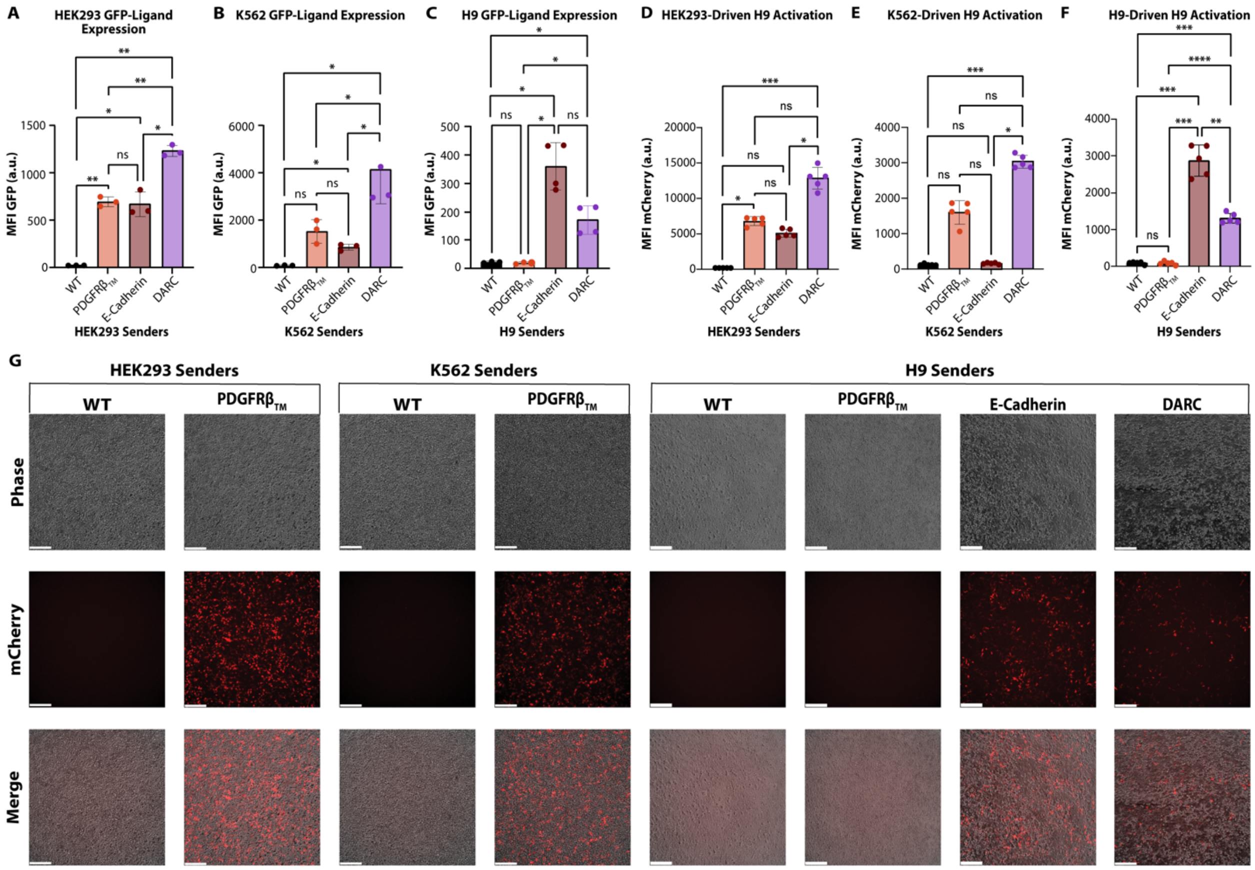

Supplemental Figure 1: GFP-ligand expression and resulting synNotch activation in different sender cell types. (A) HEK293s, (B) K562s, and (C) H9s when engineered with GFP coupled with either PDGFRβ_{TM}, Ecad, or DARC. All engineered cells are compared to respective WT cell controls. Welch's ANOVA with Tukey's multiple comparisons post hoc: ns = not statistically significant, *p<0.05, **p<0.01. mCherry H9 receivers were placed in co-culture with (D) HEK293, (E) K562, or (F) H9 senders and flow cytometry was performed using anti-mCherry immunolabeling for mCherry expression. Co-cultures with engineered sender cells were compared to respective WT cell controls (without GFP ligand). (D) and (E), Kruskal-Wallis ANOVA with Tukey's multiple comparisons post hoc: ns: not statistically significant, *p<0.05, ***p<0.001. For (F), Welch ANOVA with Tukey's multiple comparisons post hoc: ns: not statistically significant, **p<0.01, ***p<0.001, ****p<0.0001. (G) Microscopy of (D), (E), and (F) of HEK293s, K562s,

or H9s engineered with GFP coupled with either PDGFRβ_{TM}, Ecad, or DARC in co-culture with mCherry H9 receivers. Co-cultures with engineered sender cells were compared to respective WT cell control (without GFP ligand). Scale bar: 200 μm.

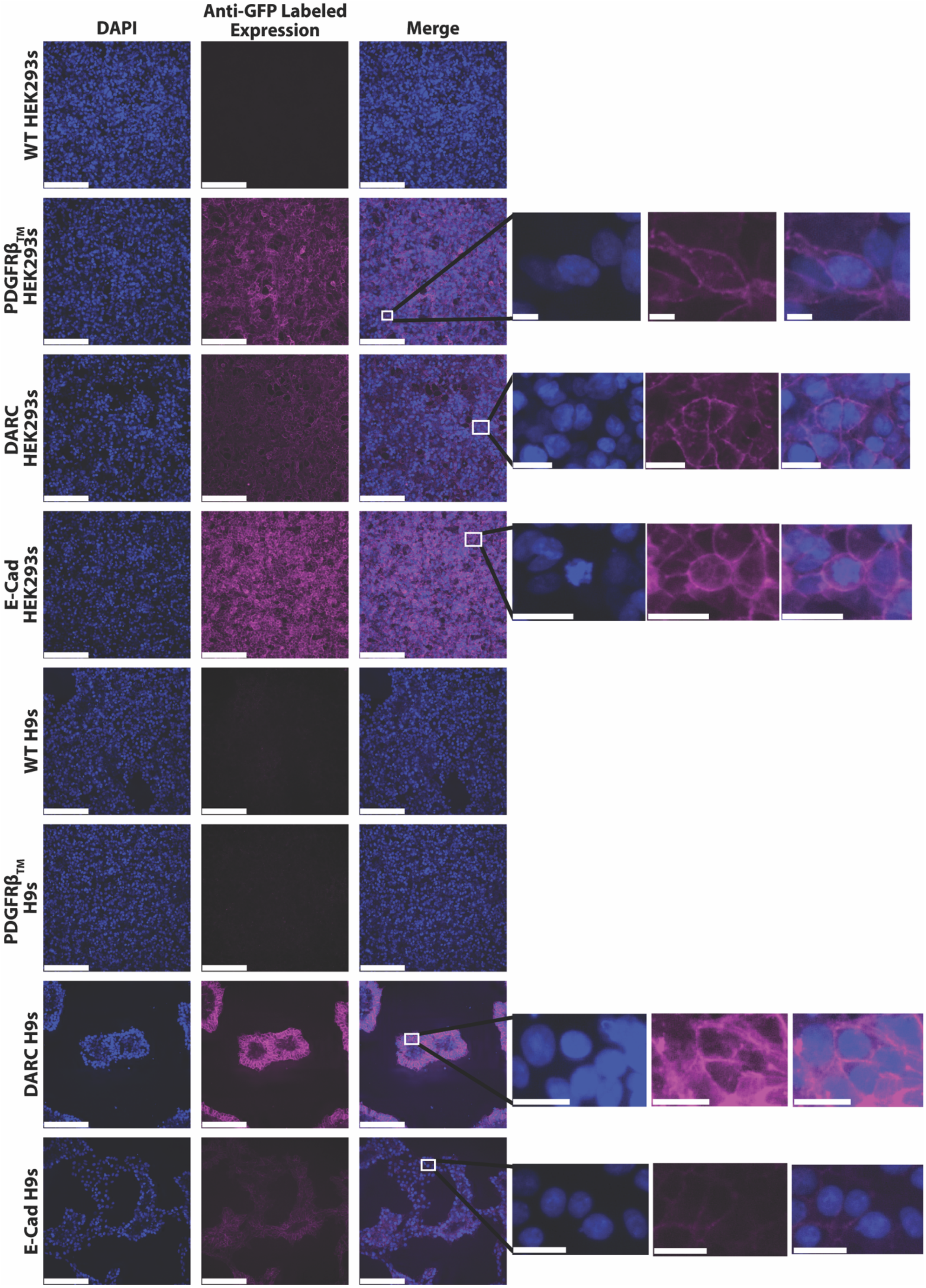
DAPI
Anti-GFP Labeled Expression
Merge
WT HEK293s
PDGFRβ$_{TM}$ HEK293s
DARC HEK293s
E-Cad HEK293s
WT H9s
PDGFRβ$_{TM}$ H9s
DARC H9s
E-Cad H9s

Supplemental Figure 2: Antibody-labeled GFP in fixed, intact HEK293s and H9 hESCs. Non-permeabilized (A) HEK293s and (B) H9s are immunolabeled for GFP. Nuclei are counterstained with DAPI. Scale bars = 200 μm or 1.25 μm for digitally zoomed insets.

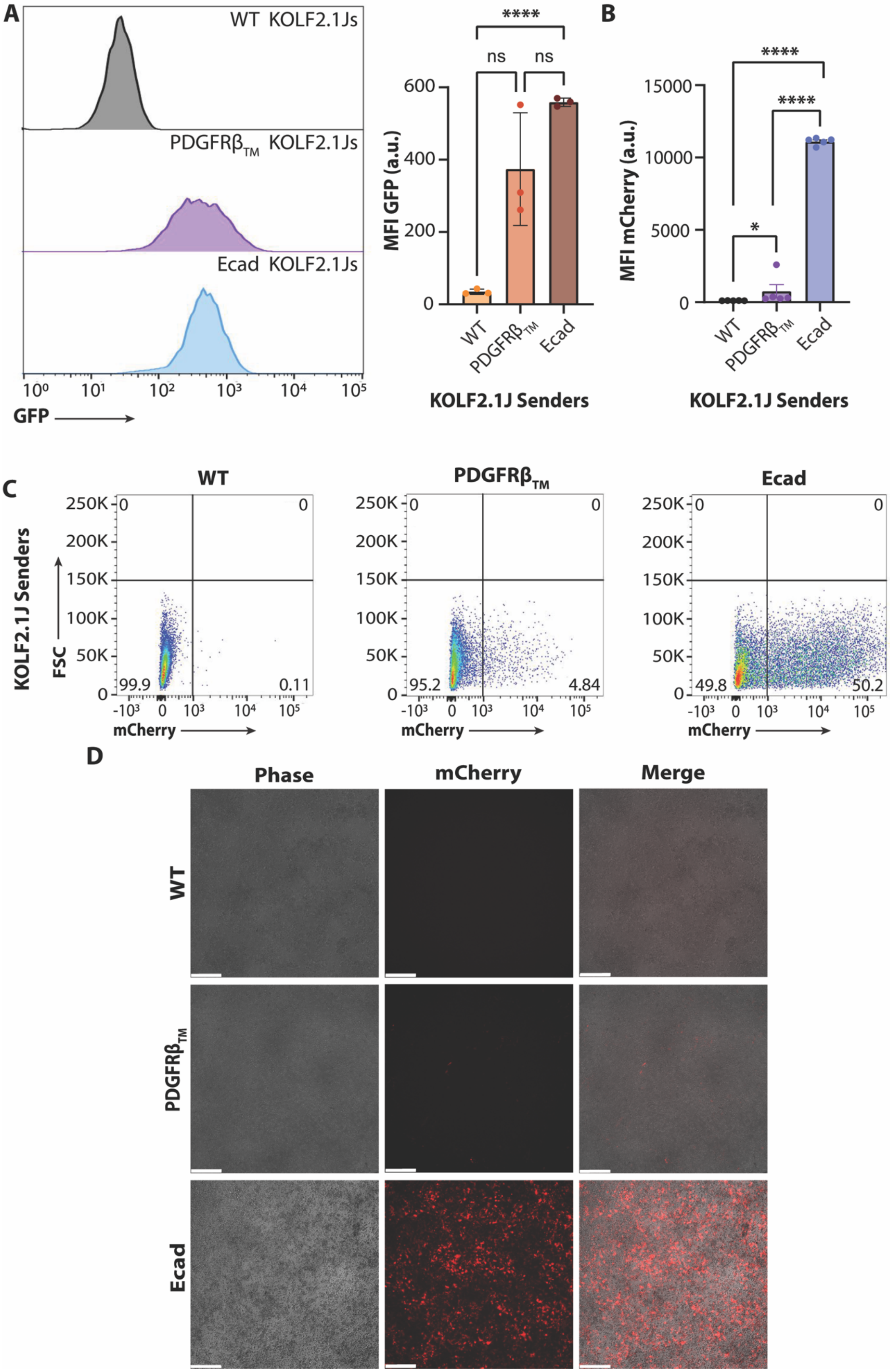
A
WT KOLF2.1Js
PDGFRβTM KOLF2.1Js
Ecad KOLF2.1Js
GFP
MFI GFP (a.u.)
ns
ns
****
WT
PDGFRβTM
Ecad
KOLF2.1J Senders
B
MFI mCherry (a.u.)
****
****
*
WT
PDGFRβTM
Ecad
KOLF2.1J Senders
C
KOLF2.1J Senders
WT
PDGFRβTM
Ecad
FSC
mCherry
99.9
0.11
95.2
4.84
49.8
50.2
D
Phase
mCherry
Merge
WT
PDGFRβTM
Ecad

Supplemental Figure 3: E-Cadherin KOLF2.1J hiPSCs activate mCherry KOLF2.1J hiPSCs in co-culture. (A) KOLF2.1J senders expressing GFP-mounted PDGFRβ_{TM} show similar levels of GFP expression compared to GFP-mounted Ecad, as shown by flow cytometry. Welch's ANOVA with Tukey's multiple comparisons post hoc: ns: not statistically significant, ****$p<0.0001$. (B-D) SynNotch co-cultures were performed by combining 50% mCherry KOLF2.1Js and 50% GFP-ligand senders (either PDGFRβ_{TM} KOLF2.1Js or Ecad KOLF2.1Js) compared to a WT control, consisting of 50% mCherry KOLF2.1Js combined with 50% WT KOLF2.1Js. An outlier assessment was performed before moving forward with Welch's ANOVA with Tukey's multiple comparisons post hoc: *$p<0.05$, ****$p<0.0001$.

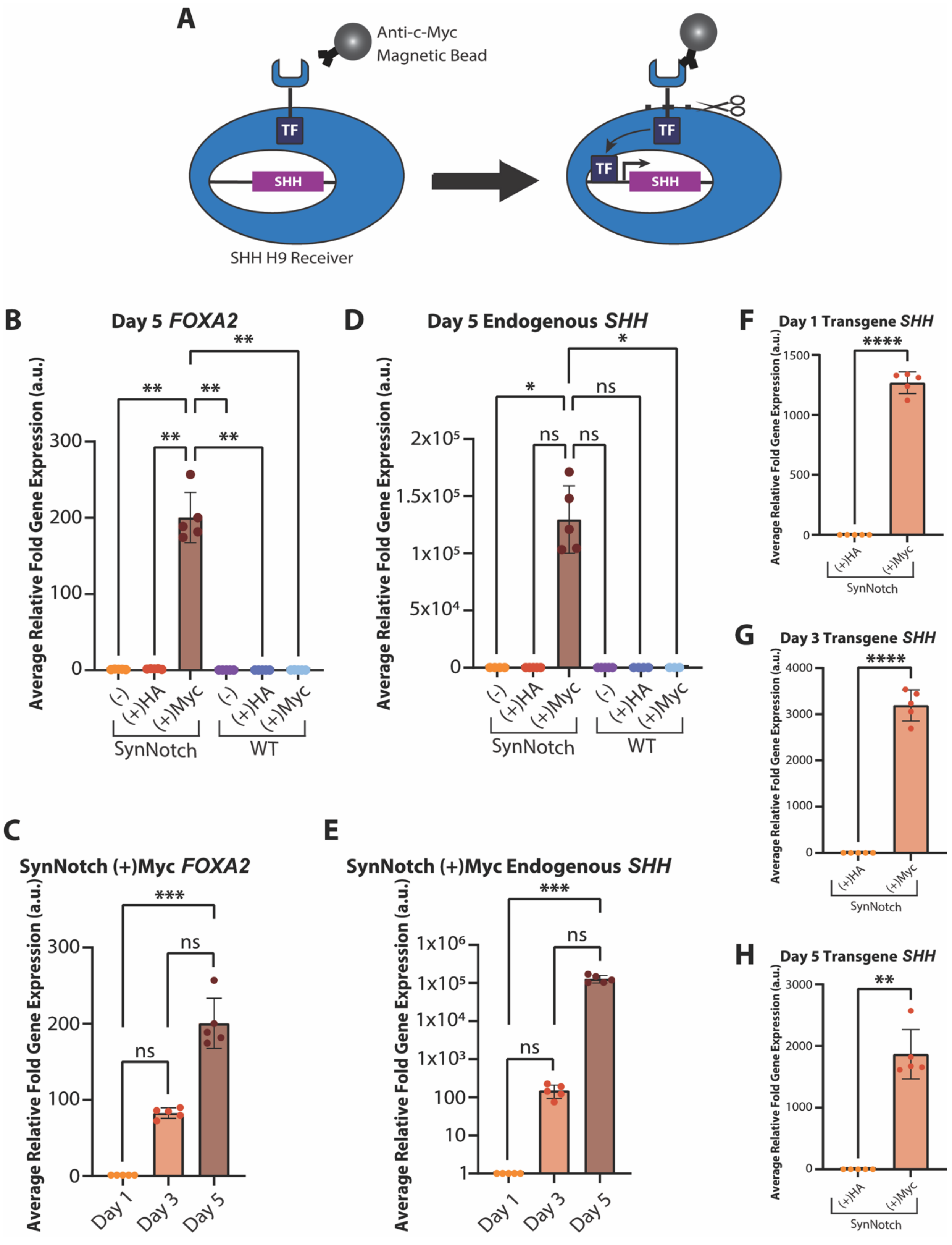
A
Anti-c-Myc
Magnetic Bead
TF
SHH
SHH H9 Receiver
B
Day 5 FOXA2
Average Relative Fold Gene Expression (a.u.)
(-)
(+)HA
(+)Myc
SynNotch
WT
C
SynNotch (+)Myc FOXA2
Day 1
Day 3
Day 5
D
Day 5 Endogenous SHH
E
SynNotch (+)Myc Endogenous SHH
F
Day 1 Transgene SHH
G
Day 3 Transgene SHH
H
Day 5 Transgene SHH
ns

Supplemental Figure 4: H9 synNotch receivers are capable of secreting SHH and inducing a floor plate-like fate. (A) SHH H9 receivers include a c-Myc epitope-tagged LaG16 receptor, capable of being activated by magnetic beads coated with anti-c-Myc antibody (anti-c-Myc beads). Recognition of the c-Myc-epitope tag by the anti-c-Myc beads leads to SHH expression by SHH H9 receivers. (B-H) After five days in culture, SHH H9s with anti-c-Myc beads ((+)Myc) were compared to SHH H9s with magnetic beads coated with anti-HA antibody (anti-HA beads) ((+)HA), which can recognize the HA-epitope tag, or SHH H9s without any beads ((-)). These results were compared against WT H9s given the same treatments. Gene expression profiling was performed via qRT-PCR to assess *FOXA2* (B and C), endogenous *SHH* (D and E), and transgene *SHH* (F-H). Transgene *SHH* refers specifically to SynNotch-driven SHH expression while endogenous *SHH* refers to native human *SHH*. For (B), Welch's ANOVA with Tukey's multiple comparisons post hoc: **$p<0.01$. For (C-E), Kruskal-Wallis ANOVA with Tukey's multiple comparisons post hoc. ns: not statistically significant, *$p<0.05$, ***$p<0.001$. For (F and G), Welch's t-test: ****$p<0.0001$. For (H), a Mann-Whitney test: **$p<0.01$.

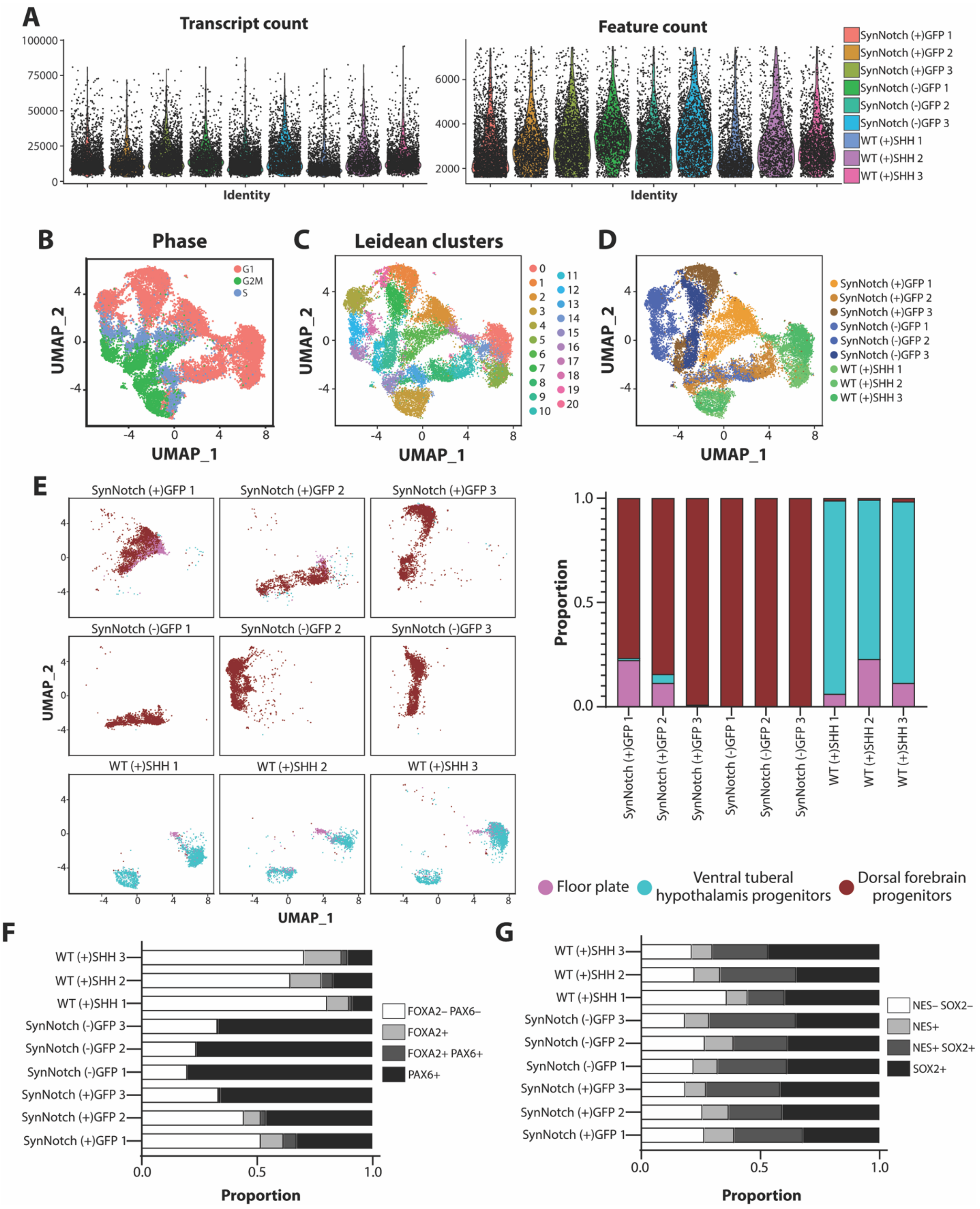

Supplemental Figure 5: Single-cell RNA sequencing quality control and cell annotation. (A) Number of detected transcripts and number of unique features per cell per sample. Each dot

represents a single cell. (B) UMAP of integrated cells, colored by imputed cell phase. (C) UMAP of integrated cells, colored by Leiden cluster. (D) UMAP of integrated cells, colored by differentiation strategy. (E) UMAP for each differentiation strategy, colored by annotated cell type for. Cell type proportions colored the same for all samples within each differentiation strategy. (F) Proportion of cell types, annotated by FOXA2 and PAX6 expression, from each individual differentiation strategy. (G) Proportion of cell types, annotated by NES and SOX2 expression, from each individual differentiation strategy.

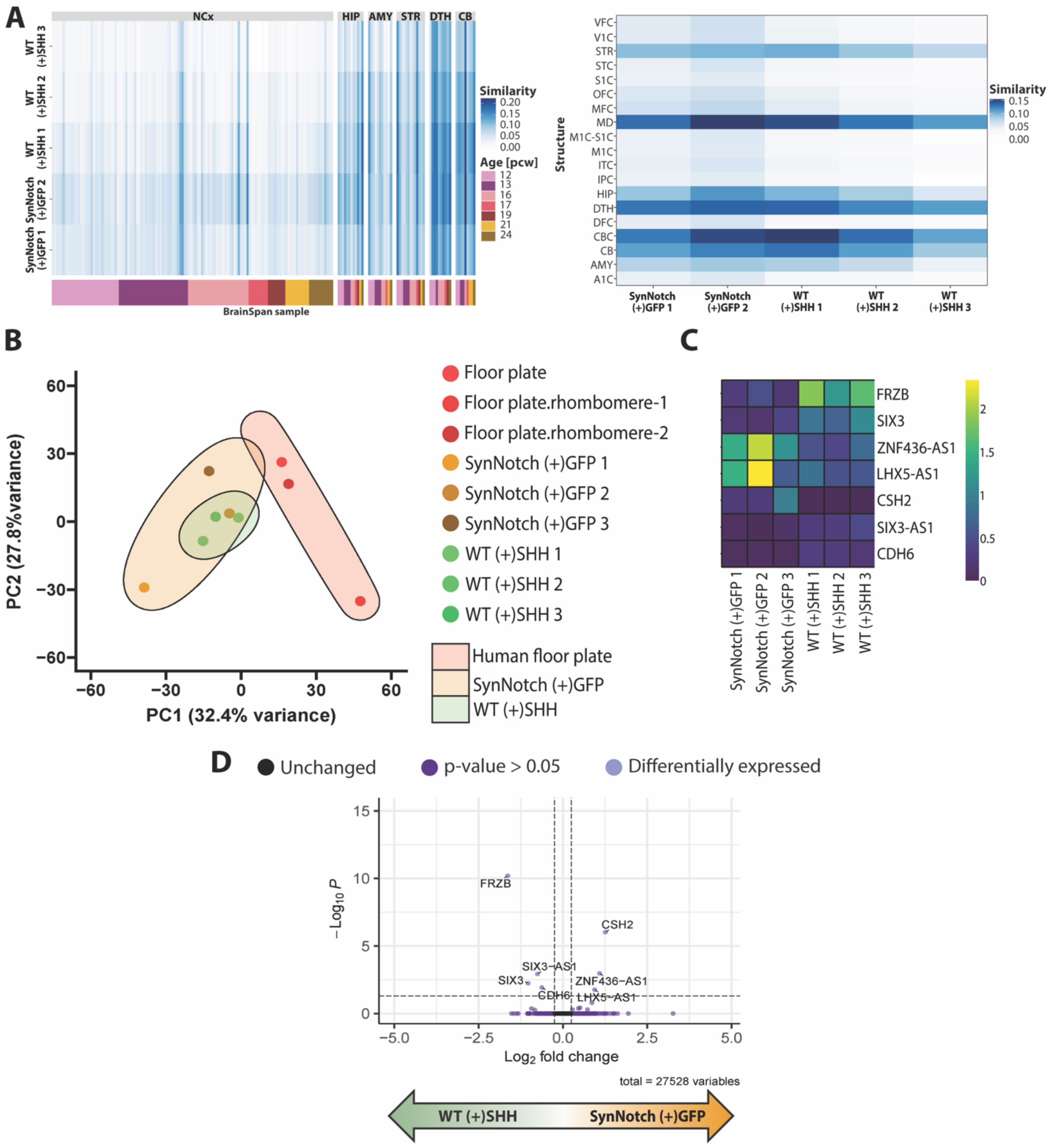

Supplemental Figure 6: Correlation of phenotypes to the BrainSpan Atlas and primary developmental human floor plate tissue with single-cell RNA sequencing data for conditions with SHH. (A) Similarity of all sequenced cells from WT (+)SHH and SynNotch (+)GFP cultures to the BrainSpan Atlas. Each plot displays a different timepoint (E11 – P56). Darker colors indicate

greater similarity. pcw – post-conception weeks; NCx – neocortex; VFC – ventrolateral prefrontal cortex; V1C – primary visual cortex; STR – striatum; STC – posterior (caudal) superior temporal cortex; S1C – primary somatosensory cortex; OFC – orbital frontal cortex; MFC – anterior (rostral) cingulate (medial prefrontal) cortex; MD – mediodorsal nucleus of thalamus; M1C-S1C – primary motor-sensory cortex; M1C – primary motor cortex; ITC – inferior temporal cortex; IPC – posteroventral (inferior) parietal cortex; HIP – hippocampus; DTH – dorsal thalamus; DFC – dorsolateral prefrontal cortex; CBC – cerebellar cortex; CB – cerebellum; AMY – amygdaloid complex; A1C – primary auditory cortex. (B) Comparison of *in vitro* floor plate-like cells derived via addition of recombinant SHH (WT (+)SHH) or synNotch-driven SHH (SynNotch (+)GFP) to *in vivo* floor plate cells from 4-6 week old embryos[2]. All populations were pseudobulked. PCA plot displaying the variability of each differentiation strategy and overlap with *in vivo* datasets. (C and D) Differentially expressed genes between floor plate cells derived from WT (+)SHH and SynNotch (+)GFP cultures. Both conditions were pseudobulked. (C) Average gene expression levels, per biological replicate, for each differentially expressed gene in floor plate cells from both conditions. (D) Differentially expressed genes computed using DESeq2. NS: not significant.

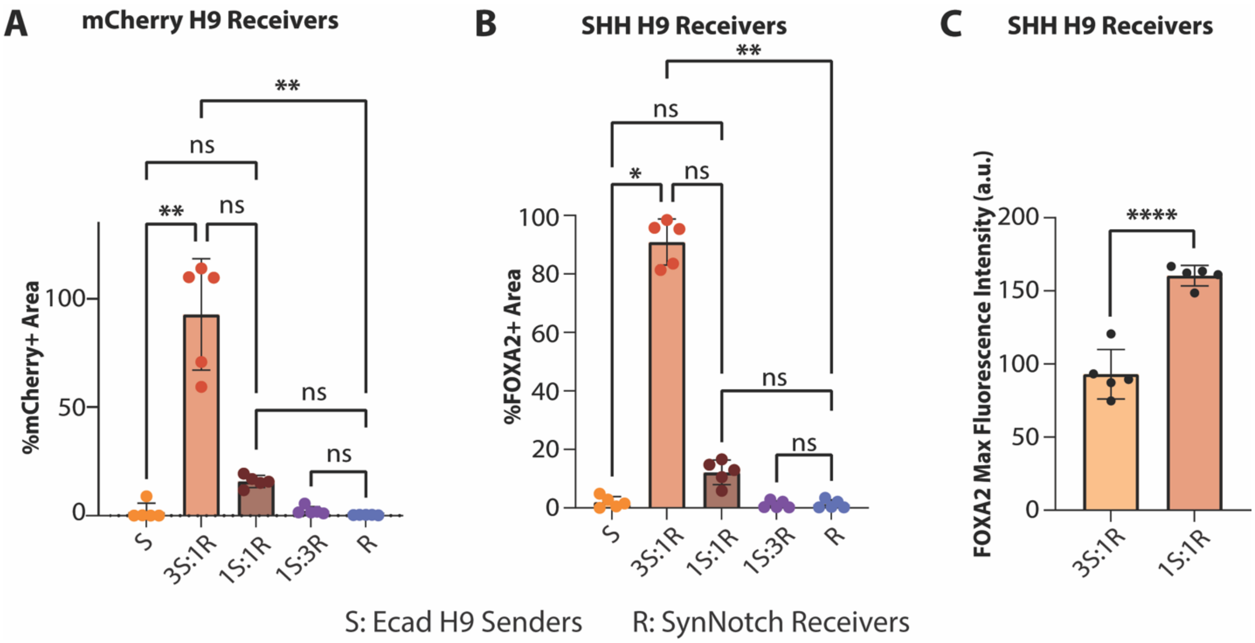

Supplemental Figure 7: SynNotch activation with varied sender:receiver ratios. (A) Ecad H9 senders and mCherry H9 receivers were placed in co-culture at varying ratios: 3:1, 1:1, and 1:3. 100% sender or receiver cells were also benchmarked against co-cultures. DAPI+ cells were assessed for %mCherry positivity from each field of view. Kruskal-Wallis ANOVA with Tukey's multiple comparisons post hoc: ns = not statistically significant, **$p<0.01$. (B) Ecad H9 senders and SHH H9 receivers were placed in co-culture at varying ratios: 3:1, 1:1, and 1:3. 100% sender or receiver cells were also benchmarked against co-cultures. DAPI+ cells were assessed for %FOXA2 positivity from each field of view. Kruskal-Wallis ANOVA with Tukey's multiple comparisons post hoc: ns = not statistically significant, *$p<0.05$, **$p<0.01$. (C) The maximum fluorescence intensity of FOXA2+ cells was calculated for Ecad H9 senders and SHH H9 receivers in co-culture at 3:1 and 1:1 ratios. Student's t-test for statistical significance: ****$p<0.0001$.

References

(1) Mátés, L.; Chuah, M. K. L.; Belay, E.; Jerchow, B.; Manoj, N.; Acosta-Sanchez, A.; Grzela, D. P.; Schmitt, A.; Becker, K.; Matrai, J.; et al. Molecular evolution of a novel hyperactive Sleeping Beauty transposase enables robust stable gene transfer in vertebrates. *Nature Genetics* **2009**, *41* (6). DOI: 10.1038/ng.343.

(2) Xu, Y.; Zhang, T.; Zhou, Q.; Hu, M.; Qi, Y.; Xue, Y.; Nie, Y.; Wang, L.; Bao, Z.; Shi, W. A single-cell transcriptome atlas profiles early organogenesis in human embryos. *Nature Cell Biology* **2023**, *25* (4), 604-615. DOI: 10.1038/s41556-023-01108-w.
